# The persistence of value-driven attention capture is task-dependent

**DOI:** 10.3758/s13414-022-02621-0

**Published:** 2023-01-06

**Authors:** A. E. Milner, M. H. MacLean, B. Giesbrecht

**Affiliations:** 1 Department of Psychological and Brain Sciences, University of California, Santa Barbara, CA USA; 2 Institute for Collaborative Biotechnologies, University of California, Santa Barbara, CA USA; 3 Interdepartmental Graduate Program in Dynamical Neuroscience, University of California, Santa Barbara, CA USA

**Keywords:** Selective attention, Attention capture, Reward, Selection history

## Abstract

Visual features previously associated with reward can capture attention even when task-irrelevant, a phenomenon known as *value-driven attention capture* (VDAC). VDAC persists without reinforcement, unlike other forms of learning, where removing reinforcement typically leads to extinction. In five experiments, factors common to many studies were manipulated to examine their impact on VDAC and its extinction. All experiments included learning and test phases. During learning, participants completed a visual search task during which one of two target colors was associated with a reward, and the other with no reward. During test, 1 week later, participants completed another visual search task in which the reward association was not reinforced. When a rewarded feature remained task-relevant (Experiment 1), VDAC was observed. When the rewarded feature was made task-irrelevant (Experiments 2–5) there was no evidence of a VDAC effect, except when the target feature was physically salient and there was a reduction in the frequency of exposure to the reward-associated feature (Experiment 5). We failed to find evidence of VDAC in Experiments 2–4, suggesting that VDAC may depend on the demands of the task resulting in vulnerability to VDAC. When VDAC was observed, extinction was also observed. This indicates that VDAC is subject to extinction as would be expected from an effect driven by reinforcement learning.

## Introduction

Selective attention is a key mechanism by which the processing of information sampled from the environment is prioritized. Models of visual attention commonly categorize the priority control scheme as either being top-down or bottom-up in nature. In this classic categorization of attentional control, top-down attention prioritizes information that is relevant to one’s behavioral goals, often via explicit knowledge, whereas bottom-up attention prioritizes physically salient or unexpected information (Corbetta & Shulman, 2002; Desimone & Duncan, 1995; Egeth & Yantis, 1997; Itti & Koch, 2000; Posner & Petersen, 1990). However, there is evidence that information processing priority is not only controlled by goals and salience, but also by factors acquired via experience that are not readily accounted for by the classic top-down/bottom-up framework (Anderson et al., 2011b; Awh et al., 2012; Chun & Jiang, 1998; Giesbrecht et al., 2013; Kasper et al., 2015; Kristjánsson & Campana, 2010). One such factor is reward history, whereby previously selected features predictive of reward magnitude and/or probability of reward bias attention, even when the features are irrelevant, not physically salient, and, importantly, *no longer predictive of reward* (Anderson et al., 2011a; Anderson & Halpern, 2017; Anderson & Yantis, 2013; Failing & Theeuwes, 2014; Hickey et al., 2010b; MacLean et al., 2016; MacLean & Giesbrecht, 2015a, b). During visual search, task-irrelevant features such as color (Anderson et al., 2011b; MacLean & Giesbrecht, 2015a), orientation (Laurent et al., 2015), and spatial location (Chelazzi et al., 2014; Cho & Cho, 2020; Liao & Anderson, 2020; Sisk et al., 2019) can guide visual attention when previously associated with reward. Typically, in these tasks, participants learn a reward association when one type of target feature (e.g., one of two target colors) predicts a high magnitude reward and the other a low magnitude reward. The associations result in faster reaction times and greater accuracy for identifying the target associated with the higher magnitude reward than that associated with the lower magnitude of reward or no reward (Anderson et al., 2011b; Hickey et al., 2010a; MacLean et al., 2016; MacLean & Giesbrecht, 2015a, b; Stankevich & Geng, 2014). However, reward associations can be distracting when they are no longer relevant to current task goals, capturing attention and impairing performance. When a previously reward-associated target feature is presented as a distractor feature in a subsequent test phase, target identification is slower and less accurate than when the reward-associated color is absent (e.g., Anderson et al., 2011a, b; MacLean et al., 2016; MacLean & Giesbrecht, 2015b). Thus, attention continues to be biased in favor of previously reward-associated features even when irrelevant and no longer predictive of reward, a phenomenon referred to as *value-driven attention(al) capture* (VDAC).

VDAC has been observed several days after reward learning in the absence of reinforcement and can resist extinction of the reward-related bias even over the course of several hundred trials (Anderson et al., 2011b; Della Libera & Chelazzi, 2009; Stankevich & Geng, 2014). Furthermore, VDAC has been shown to persist for as long as 9 months after learning the original association without any additional reinforcement (Anderson & Yantis, 2013). In the absence of reinforcement, it is expected that a previously conditioned response to a reward-predictive stimulus would cease (e.g., Pavlov, 2010), and yet, when VDAC is reported, there is often no significant reduction in the impairment over the course of test (Anderson et al., 2011b; Anderson & Yantis, 2012, 2013; Bucker et al., 2015; Failing & Theeuwes, 2014; Rothkirch et al., 2013; Sali et al., 2014; Sha & Jiang, 2016; Stankevich & Geng, 2014; Theeuwes & Belopolsky, 2012), although occasionally such an effect has been observed (Anderson et al., 2011a, 2016; Asutay & Västfjäll, 2016; Sali et al., 2018). These findings suggest that reward learning creates an unusually persistent change in attentional priority that is biased in favor of formerly reward associated features even when no longer predictive of reward. Moreover, this change in priority is highly resistant to extinction, similar to the persistent effects of spatial probabilities on attention (Geng et al., 2013; Jiang, Swallow, & Rosenbaum, 2013a; Jiang, Swallow, Rosenbaum, et al., 2013b).

The current investigation is focused on the unusual persistence of VDAC. In five experiments we investigated which task parameters affected the persistence or resistance to extinction of VDAC in the absence of reinforcement. In typical VDAC experiments reward is obtained by successfully selecting a target that is associated with reward during the learning phase. Prioritized selection during the test phase, where reward is no longer available, is due to an instrumentally conditioned response whereby habitual orienting is transferred from the learning phase to the test phase (Failing & Theeuwes, 2017). However, subsequent research has shown that VDAC is also observed via Pavlovian conditioning where no instrumental selection response is made (Bucker & Theeuwes, 2017). It remains unclear whether persistence of VDAC continues to be observed after classical conditioning or whether persistence is unique to instrumental reward learning. It is plausible that the persistence observed in many VDAC studies is amplified by the instrumental nature of the reward-associations in comparison to reward associations that are learned via classic Pavlovian conditioning. In particular, it has been demonstrated that an instrumental response alone, such as selecting a stimulus feature, can lead to prioritization of that feature, even when not associated with a reward. This phenomenon is known as *selection-driven attention capture* (SDAC; Brascamp et al., 2011; Eimer et al., 2010; Failing & Theeuwes, 2017; Kristjánsson & Campana, 2010). There is evidence that the mechanisms underlying reward and such selection-driven capture are dissociable (Kim & Anderson, 2019). However, the persistence of VDAC and SDAC were not addressed in this study so it remains unclear if persistent VDAC could be more vulnerable to extinction when learning occurs in the absence of an instrumental response.

VDAC has been replicated many times, by multiple different labs, and has even generalized across cognitive paradigms (Anderson & Yantis, 2012; Mine & Saiki, 2015). The design features of the paradigms used to induce and observe VDAC are, however, quite consistent. Specifically, the primary paradigm used to learn the reward-feature association is a visual search task containing a physically non-salient target, defined by the reward-associated feature, which is typically color. Subsequently, during the test phase (when extinction may be observed) the same visual search task is repeated but with a physically salient target feature in a different dimension than the reward-associated feature, usually a shape singleton. The presence of the now task-irrelevant reward-associated feature is then probabilistic, usually *p* = 0.5 (Anderson et al., 2011a, b; Anderson & Yantis, 2013). That homogeneity could provide both insights and limits on our understanding of VDAC, as it both facilitates the integration of evidence from different experiments but also provides little variability for assessing the boundary conditions of VDAC.

The current study was not intended to be an exhaustive catalogue of the boundaries of VDAC, its persistence, or extinction. Instead, the goal of the current study was to address the implications of typical design features of VDAC paradigms; where the relevance of the reward-associated feature, the use of salient targets, and the inclusion of absent trials are not widely discussed as having any consequence for the persistence of VDAC. Our results suggest that, indeed, such choices are not benign, particularly that of a physically salient target at test. This is important as the generalizability of VDAC effects, in the context of the present results, appears limited. Given the overwhelming homogeneity of these key features of VDAC paradigms in the published literature this issue is not trivial (see Table 1).

**Table 1:** Non-exhaustive list of value-driven attention(al) capture (VDAC) literature using paradigms with the key features of the test paradigms investigated in the current study. Excluded were ﻿﻿studies where rewards were still available at test (e.g., Bucker et al., 2015; Munneke et al., 2015), and studies showing trial-to-trial effects of reward (Hickey et al., 2010a, b, 2011). The former was excluded as the presence of rewards at test makes it unclear whether extinction learning is taking place, and the latter as trial-to-trial effects may be at least in part due to priming. It is possible that this priming is operating in the same way as VDAC resulting from extensive conditioning, but we could find no evidence that this was the case

Article	Irrelevant reward-associated feature	Physically salient target feature	Absent trials included
Anderson, Laurent & Yantis, 2011a	+	+	+
Anderson, Laurent & Yantis, 2011b	+	+	+
Anderson & Yantis, 2012	+	+	+
Anderson & Yantis, 2013	+	+	+
Bucker & Theeuwes, 2017	+	+	+
Della Libera & Chelazzi, 2009	+	-	-
Failing & Theeuwes, 2014	+	-	+
Jahfari & Theeuwes, 2017	+	+	+
Le Pelley, Pearson, Griffiths, & Beesley, 2015	+	+	+
MacLean, Diaz & Giesbrecht, 2016	+	-	+
MacLean & Giesbrecht, 2015a	+	-	+
Mine & Saiki, 2015	+	+	+
Qi, Zeng, Ding, & Li, 2013	+	+	+
Rajsic, Perera, & Pratt, 2017	+	+	+
Roper et al., 2014	+	+	+
Rutherford, O’Brien, & Raymond, 2010	+	+	+
Sali et al., 2014	+	+	+
Sali et al., 2018	+	+	+
Stankevich & Geng, 2015	+	+	-
Theeuwes & Belopolsky, 2012	+	+	+
Wang et al., 2015	+	+	-
Wang, Yu, & Zhou, 2013	+	+	+

In the present work, the task relevance of the reward-associated feature was manipulated, such that it was task-relevant at test in Experiment 1 and task-irrelevant in Experiments 2–5. The task-relevance of the reward-associated feature could play a role in the persistence of VDAC. Attention is a key component of successful learning (Jiang & Chun, 2001; Khadjooi et al., 2011) and whether a reward associated feature is to be attended (task relevant) or ignored (task irrelevant and distracting) could influence both the acquisition and persistence of the learned associations and their effects on attention capture.

The role of a physically salient target during test was also examined. Physically salient features, such as color singletons, have a robust, involuntary effect on priority (Egeth & Yantis, 1997; Folk et al., 1992; Itti et al., 1998). Shape singletons are widely used to define targets when observing VDAC during test by formerly reward-associated features that are either physically salient (Hickey et al., 2010b) or not (Anderson et al., 2011a, b; Anderson & Yantis, 2013). Consequently, it is unclear whether the persistence of VDAC may be dependent on the presence of a physically salient target. Thus, in Experiments 2 and 4, the reward-associated feature was irrelevant, and the target was not a salient singleton, but rather defined by a specific color, just like the reward-associated feature. In contrast, the target was defined as a physically salient shape singleton in Experiments 3 and 5. Finally, the role of the frequency of exposure to the reward associated feature during test was investigated. Typically, in order to initiate VDAC, the reward-associated features appear more reliably and/or more frequently during learning than during test (Anderson et al., 2011a, b; Anderson & Yantis, 2013). It is possible this asymmetry in exposure results not only in an asymmetry in learning, but also in the ability to ignore irrelevant, distracting features. In Experiments 4 and 5 absent trials where neither the previously rewarded or non-rewarded features were presented as distractors were included to assess the asymmetry in exposure between learning and test. We did not intend to manipulate these features within a single experiment but aimed to test whether a combination of task features resulted in the observation of VDAC or not and if observed was extinction also observed. See Table 2 for a summary of manipulated task features across Experiments.

**Table 2:** Summary of manipulated task features in Experiments 1–5

Experiment	Irrelevant reward-associated feature	Physically salient target feature	Absent trials included
Experiment 1	-	-	-
Experiment 2	+	-	-
Experiment 3	+	+	-
Experiment 4	+	-	+
Experiment 5	+	+	+

## Experiment 1

The main purpose of this experiment was to observe the effect of a reward-associated feature in a paradigm stripped of the stereotypical features present when persistent VDAC is observed. Specifically, the formerly reward-associated color remained task-relevant during test, just as it was during learning, and without the physically salient target and absent trials typical of many VDAC paradigms. When the reward-associated feature appears on a distractor during test (Anderson et al., 2011a, b; Anderson & Yantis, 2013), such as a feature of a stimulus that is to be *ignored*, it is possible that the intention to ignore the stimulus with the reward-associated feature impairs the acquisition of the new association during test and thus the effect of the reward-associated feature persists. It is also possible that when the reward-associated feature remains task relevant, the resolution of the conflict between the original and the new association (extinction) at test is impaired in favor of the original association, in which case reward-related effects may be more likely to extinguish when task irrelevant. In Experiment 1 the feature associated with reward continued to be task relevant. We note that because the reward-associated feature continued to be goal-relevant during the test phase that any reward related effects do not necessarily reflect attention capture because these features would continue to be prioritized in a goal-directed manner, even though reward associations were no longer reinforced.

During the learning phase, participants identified the orientation of a line segment within either a red or blue target ring, one of which was reliably followed by the receipt of a reward and the other was not. We anticipated that participants would acquire the original reward-associations during the learning phase, such that reward-associated features would be given greater priority, thus we expected to observe better performance when responding to targets with reward-associated features (faster reaction times) compared to those with features not associated with reward. During the test phase of Experiment 1, participants continued to respond to the red and blue targets and, as such, the reward-associated feature was task relevant, i.e., was to be attended. We expected that a previously reward-associated color would continue to be prioritized in visual attention compared to the non-reward associated feature. Therefore, in the case of Experiment 1, we expected faster reaction times (RTs) for discriminating targets with a reward-associated feature compared to discriminating targets that did not.

### Method

#### Participants

Participants were 27 undergraduate students (17 female, *M*_*age*_ = 19.30 years, *SD*_*age*_ = 1.41) recruited from the Psychological and Brain Sciences research participation pool at the University of California, Santa Barbara. Seven participants were excluded from the analyses because their accuracy during either learning or test was below chance thereby resulting in a final sample of 20 participants (11 female, *M*_*age*_ = 19.35 years, *SD*_*age*_ = 1.57). For all experiments, participants received course credit for their participation and monetary compensation based on performance in the learning phase (payout schedule described below). All participants reported normal or corrected-to-normal vision. All participants provided informed consent, and all procedures were approved by the University of California Santa Barbara Human Subjects Committee and the Army Research Office/Human Research Protection Office.

#### Apparatus and stimuli

All experiments were run using MATLAB R2013a and PsychToolbox, Version 3 (Kleiner et al., 2007) installed on a Mac Mini and presented on a CRT monitor (36 × 27cm) viewed at a distance of 110 cm. Stimuli in the learning and test phases were presented on a black [0, 0, 0] background. Stimuli used in the learning and test phases were six different colored rings (2.3° in diameter), centered and equally spaced on the circumference of an imaginary circle with a radius of 5° visual angle (three rings in each hemifield to the left and right of fixation). The possible colors of the rings were red [RGB: 233, 0, 0], blue [17, 103, 241], orange [186, 93, 16], teal [22, 128, 109], brown [140, 111, 78], green [63, 129, 45], gold [146, 111, 16], violet [169, 60, 203], pink [199, 40, 154], mauve [166, 97, 100], moss [122, 122, 0], and gray [115, 115, 115]. Targets were defined as being red and blue rings, only one of which was presented per trial. A white [255, 255, 255] line segment was presented inside each of the colored rings and the orientations were tilted (45° to the left or right), horizontal or vertical.

#### Procedure

Participants attended two experimental sessions separated by exactly 1 week. In the initial session participants first completed a demographic questionnaire and the behavioral inhibition system (BIS) and behavioral activation system (BAS) scales (Carver & White, 1994). Participants then performed a change detection task designed to measure visual working memory capacity. Finally, participants completed the learning phase of the visual search task. In the second session participants completed the test phase of the visual search task.

##### Visual search: Learning

**Fig. 1:**
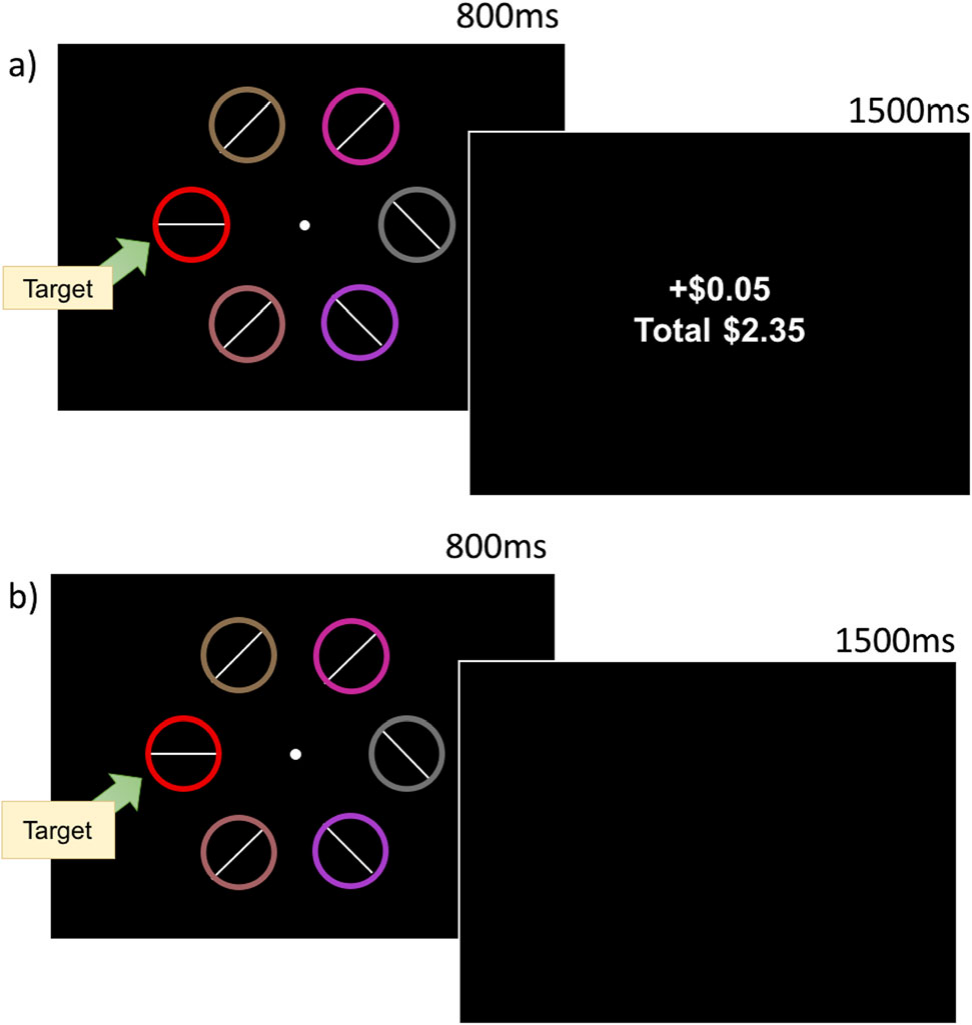
**a** Learning task (800 trials): targets were either red or blue rings and only one target color was presented per trial. Inside target rings were either a horizontal or vertical line. When the line was vertical participants responded by pressing “Z” and when horizontal “M.” One of the target colors was rewarded ($0.05) if participants responded accurately while the color target color was not rewarded. **b** Test task (1,600 trials): same task but no longer an opportunity to win rewards. The feedback display which followed the stimulus display was blank

Each trial consisted of a fixation display, a stimulus display and a feedback display. A trial began with a white fixation circle (.5° in diameter) presented in the center of the display. The duration of the fixation circle was 500, 600, 700, or 800 ms and was randomly determined on each trial. The stimulus display followed the fixation circle and consisted of a target ring (either red or blue) and five distractor rings, the colors of which were randomly drawn without replacement from the color list above (see Fig. 1a). The line segments within the distractors were orientated 45° to the left or to the right. Within the target ring the line orientation was horizontal on half the trials and vertical on the other half (distributed equally within red and blue target trials). Participants were instructed to press “Z” on a standard QWERTY keyboard if the line orientation within the target was vertical and “M” if it was horizontal, using their left and right index fingers respectively. The stimulus display was presented for 800 ms or until a response was made. Once a response was made, the feedback display was presented for 1,500 ms, which indicated the amount of money the participant had won on that trial and the total amount accrued over the course of the experiment. Participants only received a reward when a correct response was made, although this was not made explicit to the participants. The color of the target (red or blue) that predicted a reward was counterbalanced across participants. On rewarded trials participants could win $0.05 and 80% of these trials had the potential to be rewarded (if a correct response was made). On non-rewarded trials there was no possibility of reward, regardless of accuracy.

The learning phase consisted of 20 practice trials that were not rewarded, followed by ten blocks each with 80 trials, resulting in 400 potentially rewarded and 400 non-rewarded trials. Participants were informed that they had the chance to win money on each trial and that they would be paid the total amount accrued over the course of the experiment at the end of the session. Participants could win a maximum of $16 in the learning phase and on average participants were paid $13.90. Accuracy was measured as the number of correct responses to the line orientation within targets and reaction time was measured relative to the onset of the stimulus display on correct trials.

##### Visual search: Test

The stimuli and procedure were identical to the learning phase, participants continued to respond to the line orientation within the red and blue targets. However, during the test phase participants were no longer rewarded and once a response was made, instead of the 1,500 ms feedback screen, the display was blank (see Fig. 1b). There were two conditions during test: (1) “rewarded” trials, where the target color was previously associated with reward, and (2) “non-rewarded” trials, where the target color was not previously associated with reward during the learning phase, both trials contain a former target color. The test phase began with 20 practice trials, followed by 20 blocks of 80 trials, yielding 800 rewarded and 800 non-rewarded trials.

##### BIS/BAS

The behavioral inhibition system (BIS) is thought to regulate aversive motives to move away from something unpleasant. Whereas the behavioral approach system (BAS) regulates appetitive motives to orient towards things that are desirable. The BAS scale has three sub-components: (1) drive, (2) fun-seeking, and (3) reward responsiveness. Previous evidence has demonstrated that there is an association between the BAS drive component and the effect that a high magnitude reward has on attention (Hickey et al., 2010a, b).

##### Change-detection task

Previous evidence has shown that individual differences in visual working memory (VWM) capacity is associated with VDAC (Anderson et al., 2011b) such that those with greater capacity working memory capacities demonstrate less value-driven attention capture. Although not necessary to observe either the presence, or extinction of VDAC we attempted to replicate this correlation, and if present, examine whether it also affects the persistence and/or extinction of the effect. We measured each participant's VWM capacity using a change detection task (Luck & Vogel, 1997). Participants were briefly presented with a display that consisted of four or six squares (each .65°) distributed throughout the visual search space in a randomly determined pattern for 100 ms. Two-thirds of the trials were set-size 6 and the other third were of set-size 4. Following this a blank delay screen was presented for 900 ms and then another display appeared that contained only one square that occupied the same location of a square that had previously been presented. Participants indicated whether the color of the square in that location had remained the same or had changed by making an unspeeded key press (“Z” and “M,” respectively). Visual-working memory capacity was calculated using Cowan’s formula (Cowan, 2001), which is calculated separately for each set size by multiplying set size by the difference between the hit rate and the false alarm rate (Set size*(Hit rate - False alarm rate)). A weighted average was calculated across set-sizes to get an overall estimate of working-memory capacity (K). K was not measured for one participant in Experiment 4 due to experimenter error.

#### Design and analysis

Reinforcement learning and VDAC were measured by comparing RT on correct trials in the presence of reward-associated search items to that in the presence of non- rewarded targets in the learning and test phases respectively – the key, and only, difference being that during the test phase these features were no longer predictive of reward (i.e., the association was no longer reinforced). Our analyses focus on RT, as this was a speeded task and accuracy was expected to be high. Trials where RTs were less than 200 ms were removed from the analyses. For both learning and test participants accuracy data was divided into bins containing 200 trials averaged over reward condition (overall accuracy). The 95% confidence interval around chance accuracy (.5) was calculated for each time bin. Participants whose accuracy fell below the upper bound of this interval in two or more bins were performing effectively at chance and removed from the analyses. This approach was used to exclude participants across all experiments, the cut-off for exclusion ranged from 52.86–56.20% across experiments.

Our definition and calculation of VDAC, specifically as a difference between reward associated former targets and a control condition that accounts for selection history effects, is shared by many others (Bucker & Theeuwes, 2017; Della Libera & Chelazzi, 2009; Failing & Theeuwes, 2014; Le Pelley et al., 2015; Mine & Saiki, 2015; Rajsic et al., 2017; Roper et al., 2014; Theeuwes & Belopolsky, 2012; Wang et al., 2013). However others have employed a different definition (e.g., Anderson et al., 2011a, b; Qi et al., 2013; Sali et al., 2014, 2018; although no Anderson & Yantis, 2013), where VDAC is indicated by a difference in performance between trials with a reward-associated former target (present) and those without (absent). This operationalization of VDAC needs to be considered when placing our results in the context of the literature. Our calculation of VDAC is designed to exclude selection-history effects by contrasting conditions with the same degree of selection history. We address selection-driven attention capture (SDAC) effects in Experiment 4.

We analyzed our data using Bayesian generalized linear mixed effects models (GLMMs) using the stan_glmer function in the R package *rstanarm* (Goodrich et al., 2018). To determine whether the data were from a standard normal distribution we used the kstest function from the statistics and machine learning toolbox in MATLAB. All data were normally distributed across experiments. Therefore, we used the default link function in stan_glmer for normally distributed data. The default priors for the stan_glmer function were used which are weakly informative. The model structure across experiments included trial and reward condition as the fixed factors and a random effects structure with an intercept of 1 and reward condition and subject. We did not use the maximal random effects structure because it did not converge, likely because the model was overparameterized due to the inclusion of trial as a random effect. Based on a model comparison comparing four models with different random effects structures using the loo_compare function, we chose the best model, which included subject and reward condition in the random effects structure. Therefore, the following model structure was used across experiments separately for learning and for test: rt ~ trial*cond + (1 + cond|subject), where rt = reaction time in ms; trial = trial order/rank; and cond = rewarded, non-rewarded, absent (Experiments 4 and 5 only); and subject = subject number.

We used two approaches to provide information about the probability of, and evidence for (or against), possible effects. First, to provide information about the range of probable parameter estimates that received some support from the observed data, we constructed Bayesian Support Intervals (SIs; Wagenmakers et al., 2022). Bayesian SIs consider the posterior distribution and the prior distribution. In the present case, a SI was computed using a support criterion of BF = 3, which provides an interval that contains parameter estimates that are supported by a moderate amount of evidence. More specifically, it means that the SI contains only those parameter estimates that increased in probability by a factor of three based on the evidence. Second, to provide information about the strength of the evidence, we computed Bayes Factors relative to a region considered equivalent to the null hypothesis (as opposed to a point null, e.g., H_0_ = 0). The null regions (Region of Practical Equivalence, RPE) were defined by calculating the standard deviation of each parameter estimate and multiplying by ±.1, which corresponds to a small effect, which was defined as: [-.1*SD_RT_, .1*SD_RT_] for each model, as recommended by (Kruschke & Liddell, 2018) for linear models.

The Bayesian SI can then be compared to this null region. If the estimates bounded by the SI do not overlap the null region, then the effect can be considered probable. Evidence for an effect (or in favor of the null) was determined by computing the BF relative to the null region. Specifically, the reported BFs are a ratio of the change in posterior odds and change prior odds for the parameter falling inside or outside the null region. Interpretation of the BFs follows convention (Jeffreys, 1998), such that values of 1 or less indicate more evidence that there is practically no effect relative to the evidence in favor of there being an effect. As values increase above 1, there is increasing evidence in favor of the parameter falling outside the null region relative to the evidence in favor of the parameter falling inside the null region. Using these two approaches, we determine whether the effect is probable (i.e., via the SI) and the strength of the evidence for that effect or the null (i.e., via BF).

### Results

#### Visual search: Learning

**Fig. 2:**
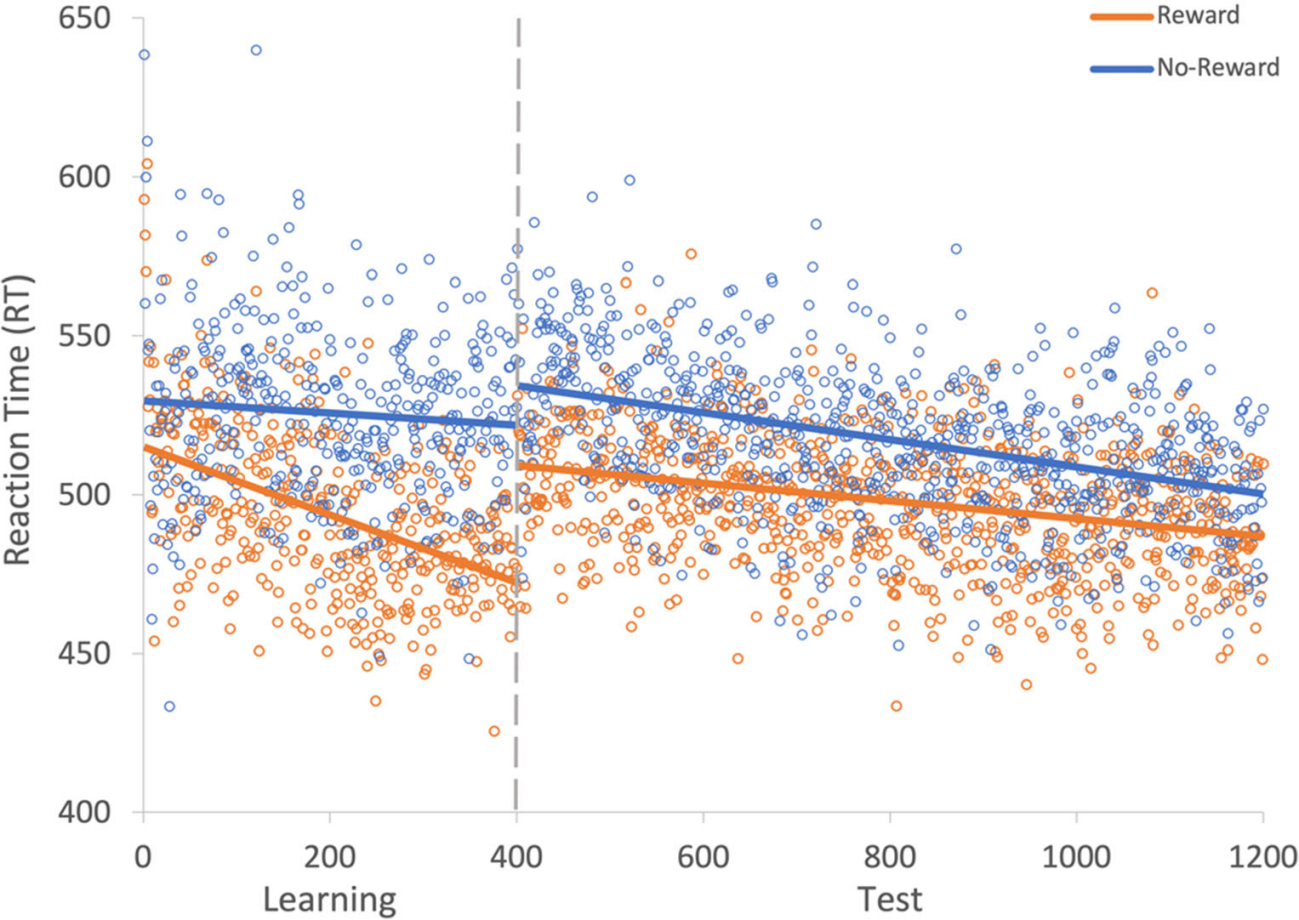
Results for both learning and the test phase of Experiment 1. Data points are the raw reaction time (RT) data averaged across participants for each trial for rewarded and non-rewarded trials separately. The regression lines are the predicted RT for rewarded and non-rewarded trials across time from the generalized linear mixed effects model (GLMM)

**Fig. 3:**
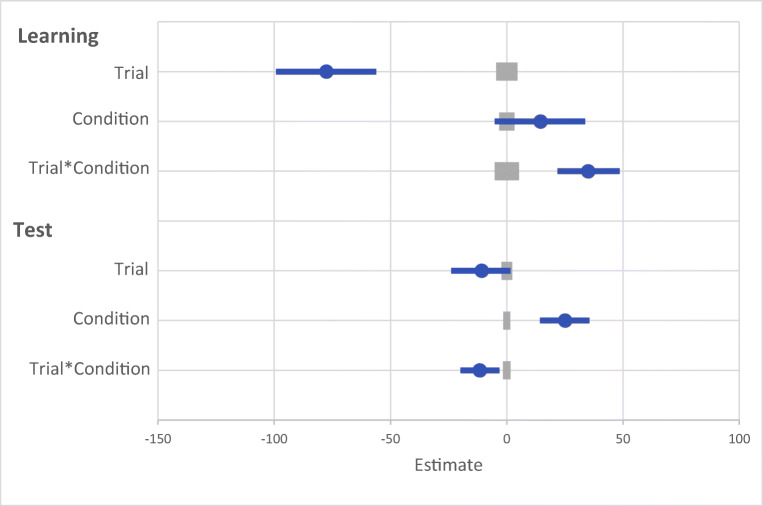
Mean point estimates and support intervals for each of the fixed effects from the generalized linear mixed effects models (GLMMs) for both learning and test in Experiment 1. Grey boxes denote the null region (region of practical equivalence, RPE)

A generalized linear mixed effects model (GLMM) was used to examine RT on correct trials with the fixed effects of reward condition (rewarded or non-rewarded target feature) and trial order (trials 1–800; see Fig. 2) during the learning phase. An effect of trial was probable, such that RTs got faster as trial order increased (*b* = -77.60, *SI *= [-99.24, -56.19], *RPE* = ± 4.59, *BF* > 1000; see Fig. 3). The effect of reward condition was not (*b* = 14.49, *SI* = [-15.14, 33.75], *RPE* = ± 3.30). Furthermore, there was substantial evidence in support of the null for the effect of reward condition (*BF =* 0.077; see Lee & Wagenmakers, 2014, p.105 for their heuristic scheme for interpreting BF_10_). However, the interaction between trial and reward condition was probable, where RT decreased at a faster rate for rewarded than non-rewarded targets during learning (*b* = 34.98, *SI* = [21.82, 48.53], *RPE* = ± 5.22, *BF* > 1000).

#### Visual search: Test

The same GLMM approach was used to examine RTs on correct trials during the test phase (see Fig. 2). During the test phase the effect of trial was not probable (*b* = -10.78, *SI* = [-23.85, 1.55], *RPE* = ± 2.33; see Fig. 3), and there was moderate evidence in support of the null (*BF* = 0.059). An effect of reward condition was probable, such that RTs to rewarded target features were faster than to non-rewarded (*b* = 25.10, *SI* = [14.25, 35.54], *RPE* = ± 1.52, *BF* = 113.80). This effect indicates that the effect of reward persisted during the test phase. An interaction between trial and reward condition was probable, such that the difference in RT to rewarded and non-rewarded target features was reduced as trial order increased (*b* = -11.64, *SI* = [-19.93, -3.19], *RPE* = ± 1.60, *BF* = 3.10), indicating that there was moderate evidence of a reduction in the effect of reward.

To assess whether the effect of reward continued to persist throughout the test phase, a Bayesian paired-samples t-test examining the difference between rewarded and non-rewarded trials was conducted on the final 100 trials of the test phase. There was very strong evidence of an effect of reward (*BF* = 86.04). This suggests that despite the reduction between rewarded and non-rewarded features throughout the test phase the effect of reward continued to persist.

##### BIS/BAS

We aimed to examine whether such individual differences in the BAS sub-scales could account for variations in the VDAC effect. Three participants were excluded from these analyses due to failure to complete the BIS/BAS questionnaire. The mean score for the BIS scale was 2.92 (*SD* = 0.50). The mean and SD for each of the BAS components were: drive (*M* = 2.97, *SD* = .57), fun seeking (*M* = 2.91, *SD* = .57), and reward responsiveness (*M* = 3.61, *SD* = .42). We correlated the BIS and the three BAS factors with VDAC (defined as the difference in response time on previously rewarded compared to previously non-rewarded features) during both learning and extinction. There was substantial evidence of a correlation between score on the BIS scale and the VDAC effect during learning (*r*(15) = .60, *BF* = 5.68) and test (*r*(15) = .54, *BF* = 3.16). Both correlations indicate that the higher a participant’s score on the BIS scale the larger their VDAC effect.^1^

##### Working memory

We correlated K (*M* = 2.90, *SD* = 1.17) and VDAC (reward vs. no reward RT). There was no evidence of a correlation (*r*(18) = .018, *BF* = 0.28). Thus, we failed to find evidence for a relationship between working memory capacity and the effect of reward on attention in Experiment 1.^2^

### Discussion

During the learning phase of Experiment 1 there was extreme evidence that participants learned the reward association, as RTs decreased at a faster rate to rewarded than non-rewarded targets over repeated exposures. During the test phase of Experiment 1, participants continued to respond to the red and blue targets that were previously associated with either reward or the absence of reward. The feature associated with reward at learning thus remained task relevant during the test phase. We observed strong evidence for an effect of reward, as indicated by faster RTs to previously rewarded target features compared to those not previously associated with reward as has previously been observed (Hickey et al., 2010b; MacLean & Giesbrecht, 2015a). However, an interaction between reward condition and trial was probable, suggesting that while the effect of reward persisted, when the reward-associated feature remains task relevant, the effect is reduced although still persistent. These results establish a point of comparison as we introduce the stereotypical VDAC paradigm features to establish their effects on the persistence, and extinction of VDAC.

## Experiment 2a

In Experiment 1 the reward-associated feature remained task relevant during test and there was evidence that VDAC persisted but was also subject to extinction. The main purpose of Experiment 2 was to examine the persistence of VDAC when the reward-associated feature was task-irrelevant, as is typical of VDAC paradigms, but again without the physically salient target feature and probabilistic reward-associated feature typical when observing VDAC during test. If VDAC persists we would expect that during the test phase the presence of a formerly reward-associated feature as a distractor would involuntarily capture attention and impair target response performance. Specifically, we expected slower RTs in the presence of a distractor with a formerly reward-associated feature compared to one with a feature not previously associated with reward.

Experiment 2 is identical to Experiment 1 except that during the test phase the colors that defined targets at learning were only ever presented as features of distractors. In this case the reward-associated feature is meant to be ignored, while different features of the same dimension (color) were meant to be attended, whereas in Experiment 1 the reward-associated feature was meant to be attended and all other colors were meant to be ignored. Not only is there a difference in whether the reward-associated feature is task relevant or irrelevant, but there is also a difference in relationship between the target defining and reward-associated features.

### Method

#### Participants

Participants were 25 students from the University of California Santa Barbara recruited from the research participation pool (12 female, *M*_*age*_ = 20.60 years, *SD*_*age*_ = 4.39). Three participants were excluded from the analyses due to poor accuracy, resulting in a final sample of 22 participants (11 female, *M*_*age*_ = 20.68 years, *SD*_*age*_ = 4.67). On average participants were paid $14.14 upon completion of the learning phase.

#### Stimuli

Stimuli were the same as Experiment 1, but moss [122, 122, 0] and gold [146, 111, 16] were not included as possible ring colors due to their similarity to green [63, 129,45] and orange [186, 93, 16], which were used as additional target colors in this experiment.

#### Procedure

Unless mentioned below, all procedures were the same as in Experiment 1.

##### Visual search: Learning

The task to respond to the line orientation within the target colors remained the same as in Experiment 1. The learning phase had two different target color sets: blue/red or orange/green. The target color pairs (red/blue or green/orange) were counterbalanced across participants.

##### Visual search: Test

**Fig. 4:**
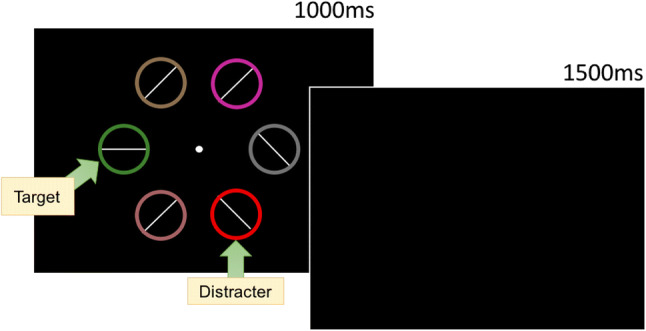
Extinction task (1,600 trials): targets at learning become distractors at extinction and the other color set that was not presented at learning become the targets at extinction

During the test phase the features that defined targets at learning (either red/blue or orange/green) were then only presented as features of distractors during test (i.e., one ring that contained a white line orientated 45° to the left or right was rendered in one of two target colors used during learning). Half of trials contained a formerly reward-associated color and the other half the color that was not formerly associated with reward. The other target color set that was not presented during learning became the new target colors during test (e.g., if a participant had red and blue targets at learning, targets at test were orange and green with red and blue circles as the critical distractors; see Fig. 4).

### Results

#### Visual search: Learning

**Fig. 5:**
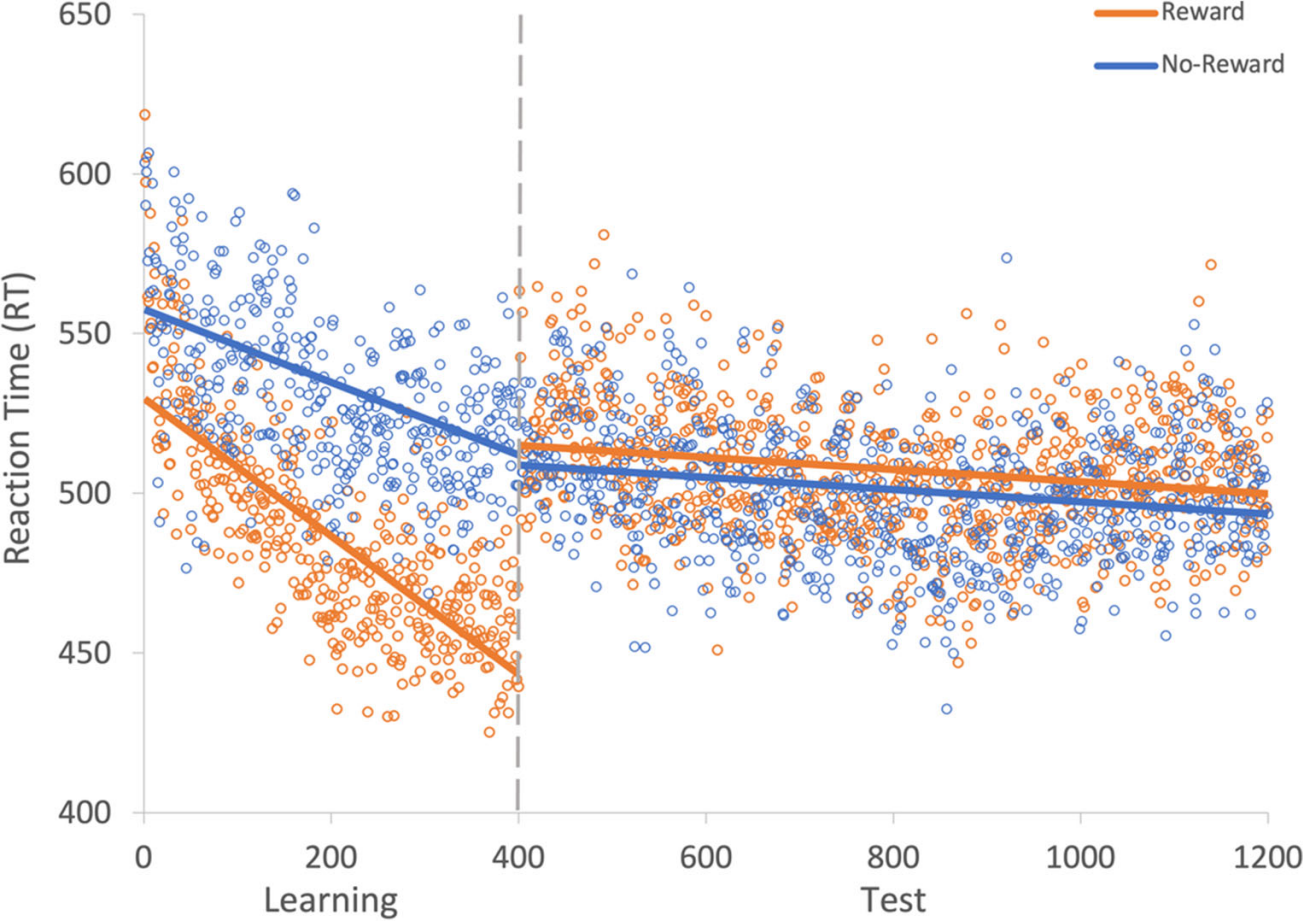
Results for both learning and the test phase of Experiment 2a. Data points are the raw reaction time (RT) data averaged across participants for each trial for rewarded and non-rewarded trials separately. The regression lines are the predicted RT for rewarded and non-rewarded trials across time from the generalized linear mixed effects model (GLMM)

**Fig. 6:**
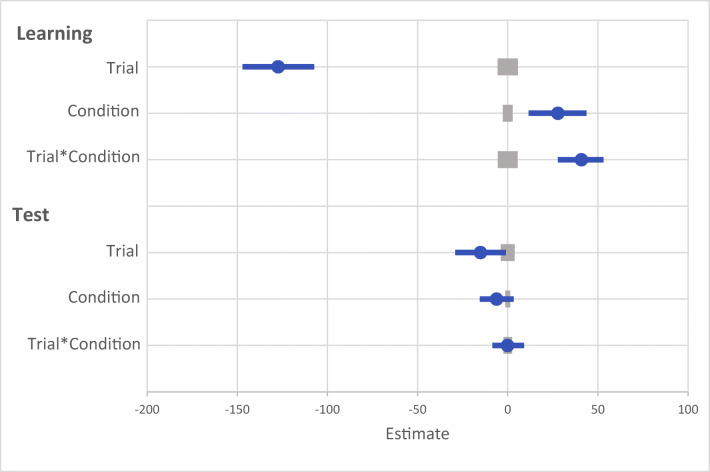
Mean point estimates and support intervals for each of the fixed effects from the generalized linear mixed effects model (GLMM) for both learning and test in Experiment 2a. Grey boxes denote the null region (region of practical equivalence, RPE)

A GLMM was used to examine RTs on correct trials during the learning phase (see Fig. 5). The effect of trial was probable, such that RTs became faster as trial order increased (*b* = -127.32, *SI* = [-147.05, -107.39], *RPE* = ± 5.57, *BF* > 1000; see Fig. 6). The effect of reward condition was also probable such that RTs were faster to rewarded than to non-rewarded target features (*b* = 27.80, *SI* = [11.61, 43.65], *RPE* = ± 2.70, *BF* = 54.92). Furthermore, the interaction between trial and reward condition was probable such that RTs decreased at a faster rate for rewarded than non-rewarded target features, indicating that participants learned the reward associations (*b* = 40.85, *SI* = [27.85, 53.14], *RPE* = ± 5.53, *BF* > 1000).

#### Visual search: Test

The same GLMM was used to examine the effect of reward during the test phase. The effect of trial was not probable (*b* = -15.15, *SI* = [-29.10, -1.07], *RPE* = ± 3.85; see Fig. 6), and there was moderate evidence in support of the null (*BF* = 0.29). The effect of reward condition was not probable (*b* = -6.21, *SI* = [-15.43, 3.27], *RPE* = ± 1.34), and there was strong evidence in support of the null (*BF* = 0.043). The interaction between reward condition and trial was also not probable (*b* = -0.12, *SI* = [-8.52, 8.96], *RPE* = ± 2.37), and there was decisive evidence in support of the null (*BF* < 0.01).

### Discussion

In Experiment 2a, during the test phase the features that defined targets during learning became features of distractors rendering these features *task-irrelevant*, as is typical when observing persistence VDAC. In Experiment 2a we again found strong evidence for reinforcement learning. However, unlike in Experiment 1, during the test phase we did not find evidence for VDAC, instead we found strong evidence for the absence of VDAC. The difference between Experiments 1 and 2a was to make the reward-associated feature task irrelevant, as such the lack of VDAC in Experiment 2a was unexpected. Due to the unexpectedness of these results, we conducted a replication of Experiment 2a with a new sample.

## Experiment 2b

The replication was identical to Experiment 2a except that the response time window was increased from 800 to 1,000 ms. We expected the longer response window to allow for greater opportunity for distraction during test.

### Method

#### Participants

Participants were 21 students from the University of California Santa Barbara recruited from the research participation pool (14 female, *M*_*age*_ = 18.85 years, *SD*_*age*_ = 1.82). Two participants were excluded because of poor accuracy resulting in a final sample size of 19 (13 female, *M*_*age*_ = 18.79 years, *SD*_*age*_ = 1.90). On average participants were paid $13.47 after the learning phase.

#### Procedure

The visual search tasks during learning and test task were identical to Experiment 2a except that the response time window was increased from 800 ms to 1,000 ms.

### Results

#### Visual search: Learning

**Fig. 7:**
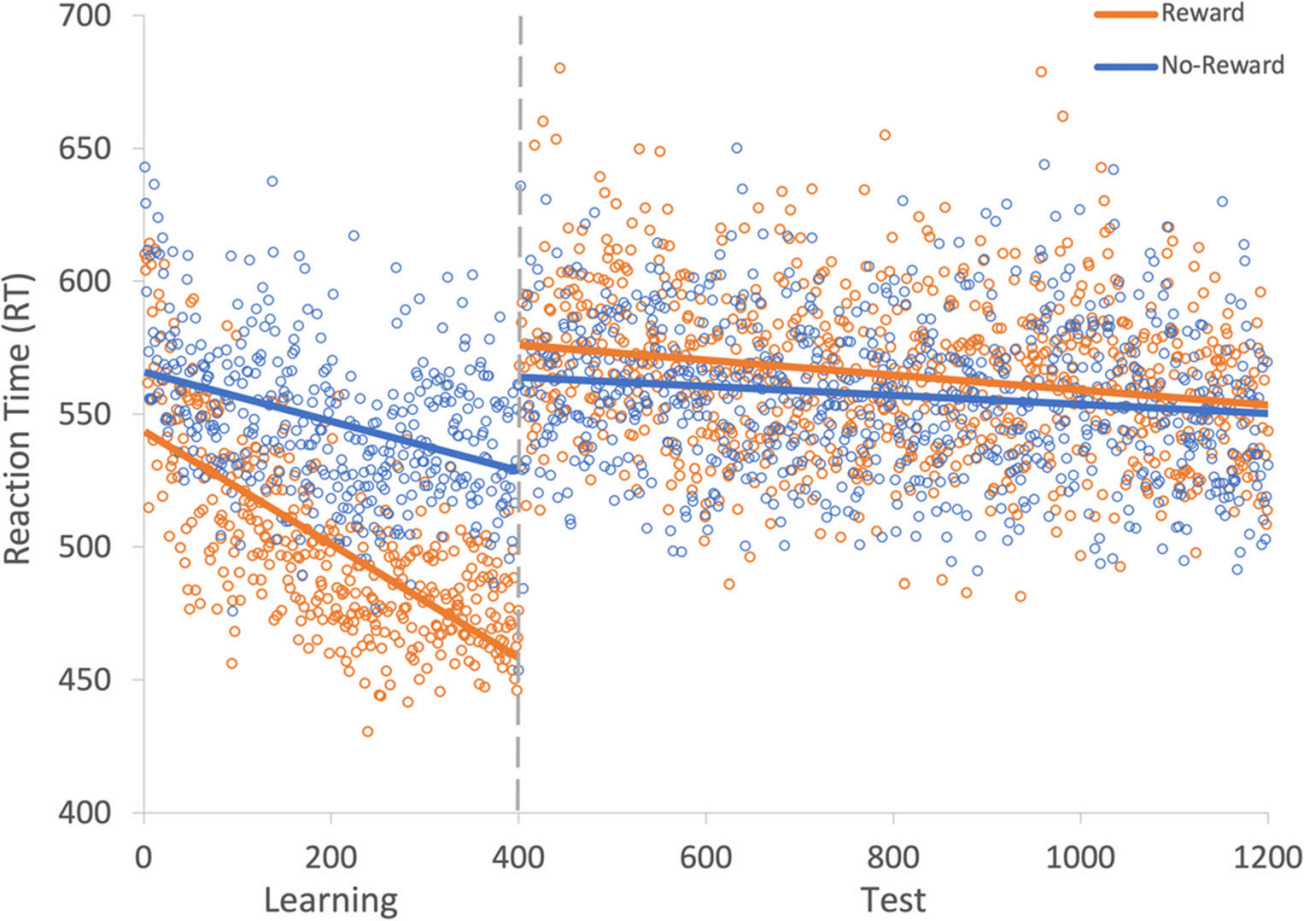
Results for both learning and the test phase of Experiment 2b. Data points are the raw reaction time (RT) data averaged across participants for each trial for rewarded and non-rewarded trials separately. The regression lines are the predicted RT for rewarded and non-rewarded trials across time from the generalized linear mixed effects model (GLMM)

**Fig. 8:**
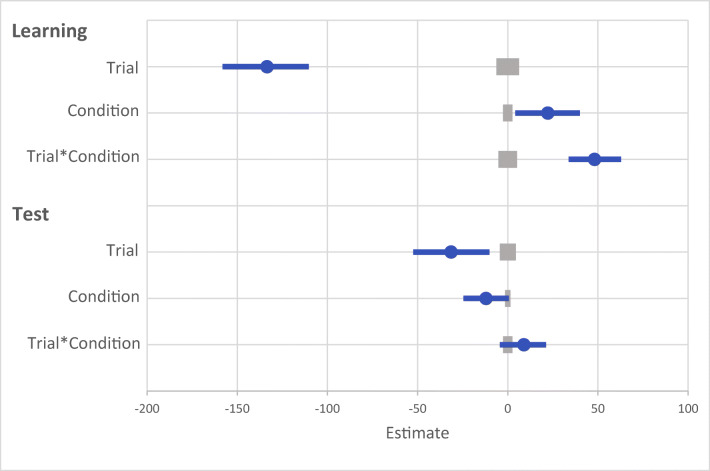
Mean point estimates and support intervals for each of the fixed effects from the generalized linear mixed effects model (GLMM) for both learning and test in Experiment 2b. Grey boxes denote the null region (region of practical equivalence, RPE)

A GLMM was used to examine RTs on correct trials during the learning phase (see Fig. 7). An effect of trial was probable such that RTs got faster as trial order increased (*b* = -133.52, *SI* = [-158.11, -110.33], *RPE* = ± 6.24, *BF* > 1000; see Fig. 8). There was anecdotal evidence of an effect of reward such that RTs were faster to rewarded than to non-rewarded target features (*b* = 22.20, *SI *= [4.12, 40.01], *RPE* = ± 2.63, *BF* = 0.94). The interaction between trial and reward condition was probable such that RT decreased at a faster rate for rewarded than non-rewarded target features (*b* = 48.14, *SI *= [33.83, 62.83], *RPE* = ± 5.11, *BF* > 1000).

#### Visual search: Test

The same GLMM was used to examine the effect of reward during the test phase. The effect of trial was probable such that participants responded faster to targets over time (*b* = -31.47, *SI* = [-52.44, -10.20], *RPE* = ± 4.43, *BF* = 5.24; see Fig. 8). The effect of reward condition was not probable (*b* = -12.07, *SI* = [-24.54, 0.57], *RPE* = ± 1.46), and there was anecdotal evidence in support of the null (*BF* = 0.44). The interaction between reward condition and trial was also not probable (*b* = 8.95, *SI* = [-4.42, 21.19], *RPE* = ± 2.64), and there was strong evidence in support of the null (*BF* < 0.01).

### Discussion

In Experiments 2a and 2b, where the reward-associated feature was task-irrelevant, we failed to find evidence for VDAC at test. Furthermore, in Experiment 2a there was strong evidence, and in Experiment 2b anecdotal evidence in support of the null, i.e., evidence for the lack of VDAC. It is possible that the longer response time window of 1,000 ms in 2b, compared to 800 ms in 2a, accounts for the discrepancy in the strength of the evidence for the null. Thus, we have mixed evidence for the absence of VDAC when the reward-associated feature is task-irrelevant. This was unexpected, but it points to two possibilities for the conditions under which VDAC does and does not occur. First, it is possible that when the reward-associated feature is task-irrelevant, as is typical, but is also in the same dimension as the new target feature (i.e., they are both colors) VDAC is suppressed. Second, it is also possible that without other common features of VDAC paradigms, such as the inclusion of a physically salient target or absent trials, VDAC is suppressed. We investigated these two possibilities in Experiments 3 through 5. We should note that these possibilities are not mutually exclusive and may even interact.

## Experiment 3

Typically, in VDAC experiments, to make the formerly reward-associated feature task-irrelevant, the target feature during test was a physically salient feature from an orthogonal feature dimension (i.e., shape vs. color; see Table 1), unlike in Experiment 2a/b where the target feature during test was the same dimension as the reward-associated feature. That difference amounts to a difference in selection demands of the test task: whether the target could be selected as the most salient feature within a dimension (e.g., shape singleton), or whether it required selection of some features in a dimension and not others without the benefit of a physically salient target feature (as in Experiments 2a/b). Physically salient features compete strongly with other factors to drive attention, including voluntary attention to relevant features which are also physically salient (Theeuwes et al., 1998). Likely, a physically salient target results in a “pop out” effect (Brascamp et al., 2011), whereby the target is automatically prioritized for attention. The persistence of VDAC may be dependent on the presence of a physically salient target when the target defining feature is in an orthogonal feature dimension to that of the reward-associated one.

For example, it is possible that when search is primarily driven by involuntary attention to a physically salient target, involuntary attention to other salient features, as is suggested to be the case with VDAC, is facilitated (ie., singleton search; Bacon & Egeth, 1994; Connor et al., 2004; Lamy et al., 2006; Lamy & Egeth, 2003). Furthermore, as the task-irrelevant feature was in a different feature dimension than the target this may complicate the competition between two salient features for attention.

In Experiment 3 we included a salient target shape to assess whether the salience and orthogonality (relative to the reward-associated feature dimension) of the target feature would influence the persistence and/or extinction of VDAC. Previous work has already produced evidence that VDAC can persist when the target defining feature at test is orthogonal, but not physically salient (MacLean & Giesbrecht, 2015a, b; target was defined as letters amongst numbers, reward associated feature was color). The following experiment focuses on the combination of salience and orthogonality, a combination that is frequently seen in VDAC literature (see Table 1), specifically whether that combination alone is sufficient to produce VDAC when the reward-associated feature is task-irrelevant.

Experiment 3 was identical to Anderson and Yantis (2013) except that there were no absent trials included at test and there were twice as many exposures to the reward-associated feature at test as there were during learning. During the test phase the target was a salient shape singleton and critical distractors could be one of the previously selected target colors at learning.

### Method

#### Participants

Participants were 25 students from the University of California Santa Barbara recruited from the research participation pool (18 female, *M*_*age*_ = 18.41 years, *SD*_*age*_ = 1.10). Eight participants were excluded from the analyses due to poor accuracy resulting in the final sample of 17 participants (15 female, *M*_*age*_ = 18.44 years, *SD*_*age*_ = 1.15). On average participants were paid $13.53 after the learning phase.

#### Procedure

##### Visual search: Learning

The learning phase of the experiment was identical to Experiment 1 (see Fig. 1a).

##### Visual search: Test

**Fig. 9:**
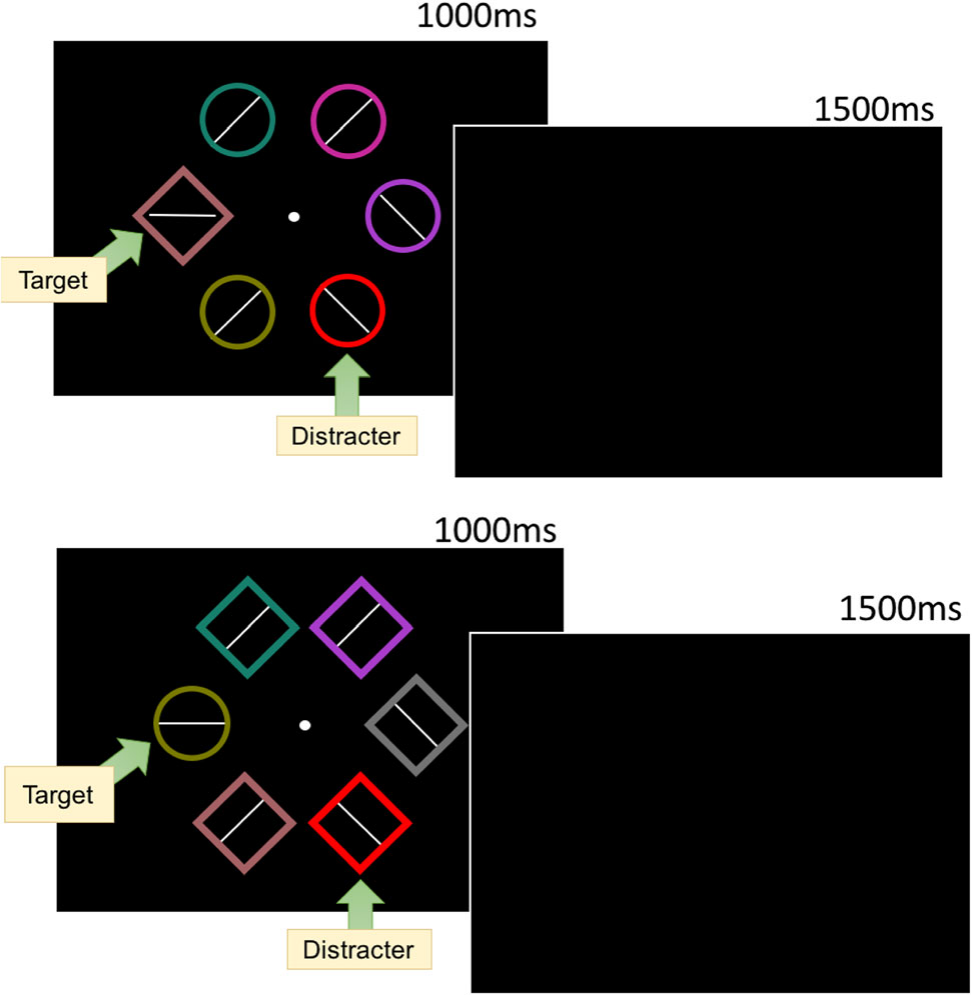
**a** Example of diamond target among circles with a critical distractor present during test. **b** Circle target among diamonds with a critical distractor present during test

The test phase was identical to Experiment 1 except that the target was defined by shape not color. Participants were instructed to search for the unique shape in the stimulus display. The unique shape could either be a diamond among circles (Fig. 9a) or a circle among diamonds (Fig. 9b). Participants responded to the line orientation inside the shape, regardless of whether the shape was a circle or a diamond. The color of the shape singleton was randomly selected from the color list without replacement and was never red or blue.

### Results

#### Visual search: Learning

**Fig. 10:**
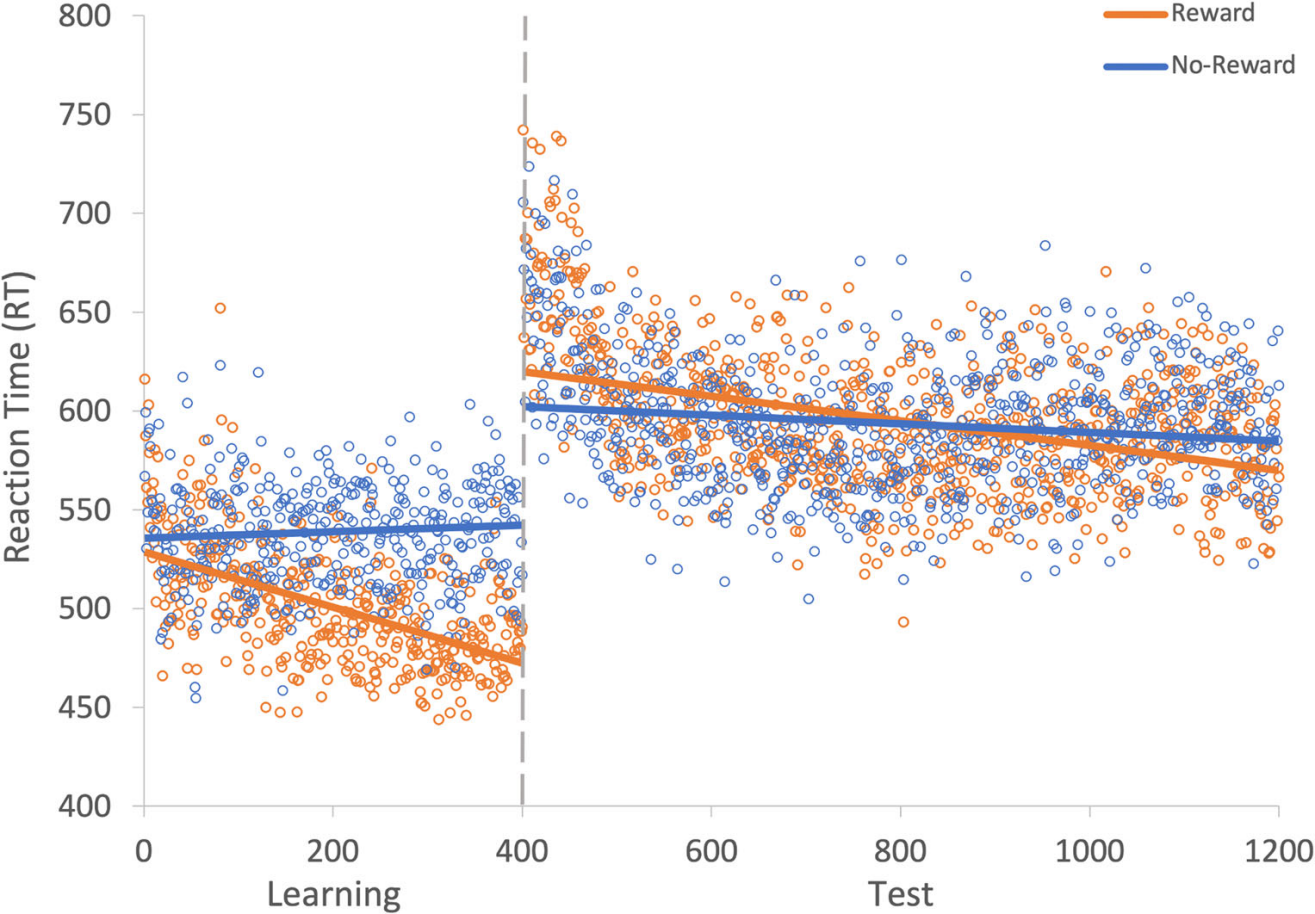
Results for both learning and the test phase of Experiment 3. Data points are the raw reaction time (RT) data averaged across participants for each trial for rewarded and non-rewarded trials separately. The regression lines are the predicted RT for rewarded and non-rewarded trials across time from the generalized linear mixed effects model (GLMM)

**Fig. 11:**
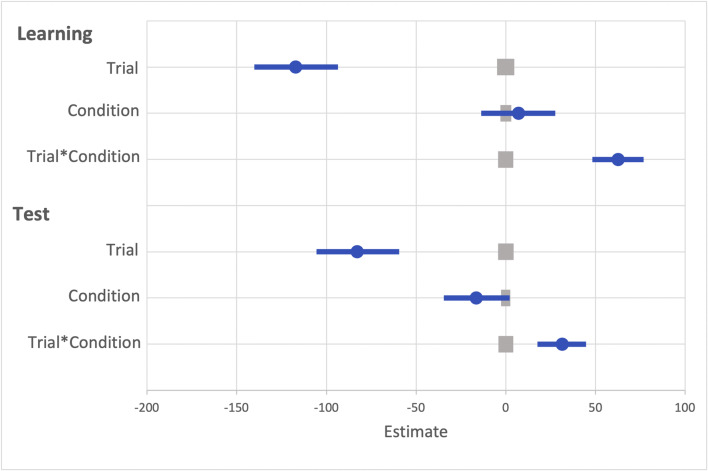
Mean point estimates and support intervals for each of the fixed effects from the generalized linear mixed effects model (GLMM) for both learning and test in Experiment 3. Grey boxes denote the null region (region of practical equivalence, RPE)

A GLMM was used to examine RTs on correct trials during the learning phase (see Fig. 10). The effect of trial was probable such that RTs became faster as trial order increased (*b* = -117.17, *SI* = [-140.13, -93.64], *RPE* = ± 4.79, *BF* > 1000; see Fig. 11). The effect of reward condition was not probable (*b* = 7.07, *SI* = [-13.72, 27.51], *RPE* = ± 2.99), and there was strong evidence for the null (*BF* = 0.019). However, the interaction between trial and reward condition was probable such that RT decreased at a faster rate for rewarded than non-rewarded target features, indicating that they learned the reward associations (*b* = 62.63, *SI* = [48.20, 76.80], *RPE* = ± 4.25, *BF* > 1000).

#### Visual search: Test

A similar GLMM was used to examine RT on correct trials during the test phase (see Fig. 10). The effect of trial was probable such that RTs got faster as trial order increased (*b* = -82.90 *SI* = [-105.50, -59.44], *RPE* = ± 4.40, *BF* > 1000; see Fig. 11). The effect of reward condition was not probable (*b* = -16.48, *SI* = [-34.63, 2.06], *RPE* = ± 2.57), and there was strong evidence for the null (*BF* = 0.13). The interaction between reward condition and trial was probable such that the difference in RT between rewarded and non-rewarded was reduced as trial order increased (*b* = 31.49, *SI* = [17.58, 44.77], *RPE* = ± 4.14, *BF =* 536.88).

The interaction between reward and trial could indicate that VDAC was present at the beginning of the text phase but was extinguished. To assess this possibility VDAC a Bayesian paired-samples t-test examining the difference between rewarded and non-reward was conducted on the first 100 trials of the test phase. There was no evidence of an effect of reward (*BF* = 0.37).

### Discussion

In Experiment 3 we found that there was no overall VDAC effect. However, there was evidence of an interaction between rewarded and non-rewarded trials which suggests that there is a change between rewarded and non-rewarded trials over time. Numerically, participants were slower on rewarded compared to non-rewarded trials at the beginning of the test phase and became faster on rewarded relative to non-rewarded trials over time. One possible explanation for this finding is that when participants engage in singleton detection search mode, the interference by the reward-associated feature is suppressed over time. It has recently been shown that attention capture by salient stimuli can be prevented by inhibitory processes (Cosman et al., 2018; Gaspelin & Luck, 2018). Furthermore, it has been demonstrated that locations that are likely to contain a salient distractor are learned to be suppressed compared to locations with a lower probability of a distractor occurring (Wang & Theeuwes, 2018). We suggest that the reward-associated distractor creates an enhanced priority signal relative to non-rewarded stimuli, but over time participants actively suppress the reward-associated distractor resulting in faster RTs to rewarded trials over time compared to non-rewarded trials (See Fig. 8). When utilizing singleton detection mode, the priority signal for any salient item in the display is increased, requiring suppression of the reward-associated distractor to avoid distraction. This interaction was not observed in experiments in which a salient singleton was not present.

The evidence of an interaction is inconsistent with previous research reporting both a main effect of reward and a lack of extinction of VDAC in the absence of reinforcement when using a very similar paradigm including a physically salient target feature orthogonal to the reward-associated feature but without absent trials (Anderson et al., 2011b; Anderson & Yantis, 2013). However, Experiment 3 included no absent trials which could have resulted in the absence of VDAC due to a higher frequency of exposure to the reward-associated feature (the reward associated feature was presented on 50% of trials compared to only 25% of trials in experiments with no absent trials).

## Experiment 4

We found evidence for the decrease in the effect of a reward-associated distractor in Experiment 3 when the formerly reward-associated feature was task irrelevant, and the target was a physically salient shape singleton as is typical when observing persistent VDAC (see Table 1). This indicates that the salience/orthogonality of the target feature at test may contribute to the effect that an irrelevant reward associated feature has on attention; a factor that has not been discussed extensively in the existing VDAC literature (although see MacLean & Giesbrecht, 2015a). In this experiment, we investigate one more typical feature of VDAC paradigms, the inclusion of absent trials, that is trials where no reward-associated feature is present as a distractor. Although the inclusion of absent trials is included as a convenient control condition to better capture the VDAC effect at test, it is possible that the inclusion of absent trials, much like the choice of a salient/orthogonal target, may in fact be key to the persistence of VDAC.

Control over attention, i.e., the ability to ignore distractors, is affected by the frequency of those distractors such that infrequent distractors are less effectively ignored than frequent ones (Geyer et al., 2008; Müller et al., 2009). It is possible that in addition to impairing the learning required for extinction, the reduced probability of the appearance of the reward-associated feature affects the ability to ignore the formerly reward-associated feature, allowing VDAC to persist.

In Experiment 4, during the test phase, we replicated the design of Experiment 2b but also included an absent condition whereby neither a previously rewarded nor previously selected target was presented as a feature of a distractor. This third condition had the additional benefit of allowing us to examine both VDAC (difference in performance between previously rewarded and non-rewarded features) and selection-driven attention capture (SDAC; difference in performance between trials with a non-rewarded previously *selected* feature and absent trials with no previously selected feature present) separately.

### Method

#### Participants

Participants were 23 students from the University of California Santa Barbara recruited from the research participation pool (14 female, *M*_*age*_ = 19.04 years, *SD*_*age*_ = 2.16). Four participants were excluded from the analyses because their performance was below chance, resulting in a final sample of 19 participants (12 female, *M*_*age*_ = 19.20 years, *SD*_*age*_ = 2.28). Participants had the opportunity to win a maximum of $8 during the learning phase and on average were paid $6.68.

#### Procedure

##### Visual search: Learning

The learning task was identical to that used in Experiment 2b, except participants were given 50 practice trials, and a reduced number of learning trials – 200 rewarded and 200 non- rewarded trials compared to 400 of each in Experiment 2b. The inclusion of equally probable absent trials during the test phase meant that the total number of trials would have doubled as compared to Experiments 1–3. This would have made the time to complete the task unfeasibly long and exacerbated confounding issues of fatigue. For that reason, we halved the number of trials during the test phase in Experiment 4 as compared to that in Experiments 1–3, resulting in the same number of trials overall with the addition of absent trials. This also meant that we needed to halve the number of learning trials, in order to have twice as many presentations of the previously reward associated distractor during test, as there were exposures during learning – an important design feature for examining persistence of VDAC during test.

##### Visual search: Test

During the test phase, features that were targets at learning became distractors at test, as in Experiment 2b. However, there was a third type of condition where no previous target colors were presented as a feature of a distractor (absent trials). The test phase began with 20 practice trials, followed by 20 blocks each with 80 trials, yielding 400 rewarded trials, 400 non-rewarded trials and 800 absent trials. Thus, as is typical of value-driven capture paradigms, the probability of a former target feature appearing was 1.00 and .50 for learning and test respectively (Anderson et al., 2011a, b; Anderson & Yantis, 2013), unlike in Experiments 1, 2a, and 2b where the probability was 1.00 for both learning and test.

### Results

#### Visual search: Learning

**Fig. 12:**
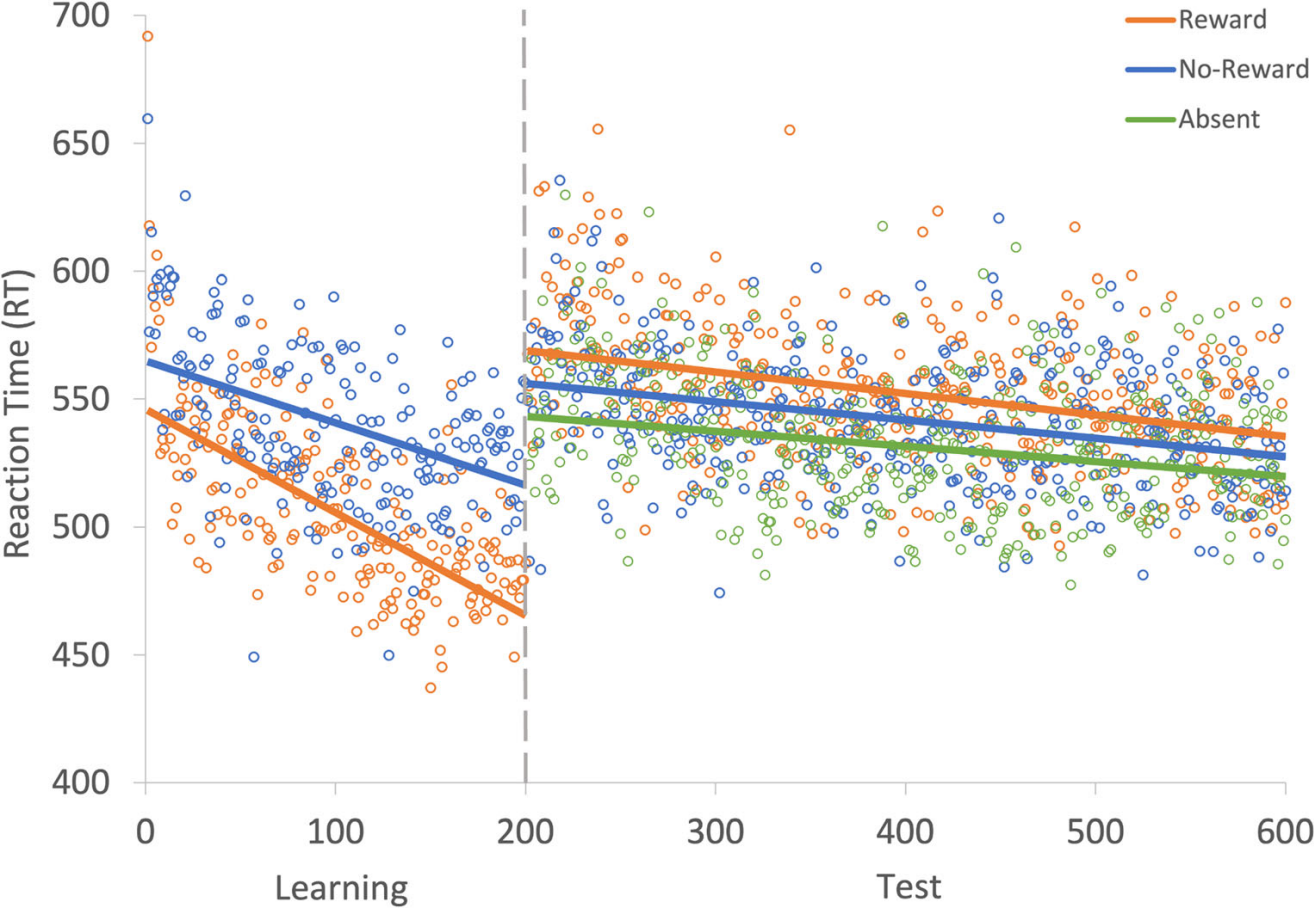
Results for both learning and the test phase of Experiment 4. Data points are the raw reaction time (RT) data averaged across participants for each trial for rewarded and non-rewarded trials separately. The regression lines are the predicted RT for rewarded and non-rewarded trials across time from the generalized linear mixed effects model (GLMM)

**Fig. 13:**
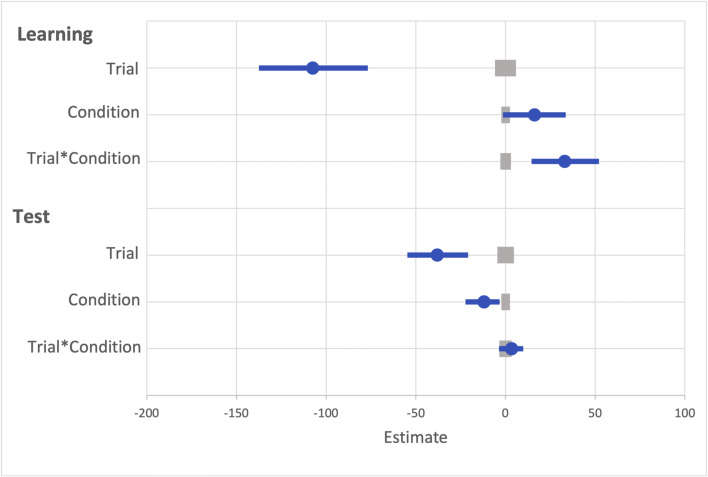
Mean point estimates and support intervals for each of the fixed effects from the generalized linear mixed effects model (GLMM) for Experiment 4 in both learning and test. Grey boxes denote the null region (region of practical equivalence, RPE)

Another GLMM was used to examine RTs on correct trials during the learning phase (see Fig. 12). The effect of trial was probable such that RTs got faster as trial order increased (*b* = - 107.55, *SI* = [-137.64, -76.79], *RPE* = ± 5.84, *BF* > 1000; see Fig. 13). The effect of reward condition was not probable (*b* = 16.18, *SI* = [-1.43, 33.58], *RPE* = ± 2.45), and there was strong evidence in support of the null (*BF* = 0.15). However, the interaction between trial and reward condition was probable such that RT decreased at a faster rate for rewarded than non-rewarded targets, indicating that they learned the reward associations (*b* = 32.92, *SI* = [14.50, 52.07], *RPE* = ± 2.93, *BF* > 1000).

#### Visual search: Test

A similar GLMM was used to examine RTs on correct trials during the test phase (see Fig. 12). The effect of trial was probable such that RTs got faster as trial order increased (*b* = - 38.17, *SI* = [-54.70, -20.99], *RPE* = ± 4.57, *BF* = 381.48; see Fig. 13). There was anecdotal evidence for an effect of reward (*b* = -12.07, *SI* = [-22.41, -3.29], *RPE* = ± 2.46, *BF* = 1.22), but the interaction between trial and reward condition was not probable (*b* = 3.37, *SI* = [-3.68, 9.84], *RPE* = ± 3.41), and there was decisive evidence in support of the null (*BF* = 0.007).

To compare the three levels of condition (rewarded, non-rewarded, and absent) we conducted three additional GLMMs to compare reward versus no-reward, no-reward versus absent, and reward versus absent conditions.

##### Reward versus no-reward

**Fig. 14:**
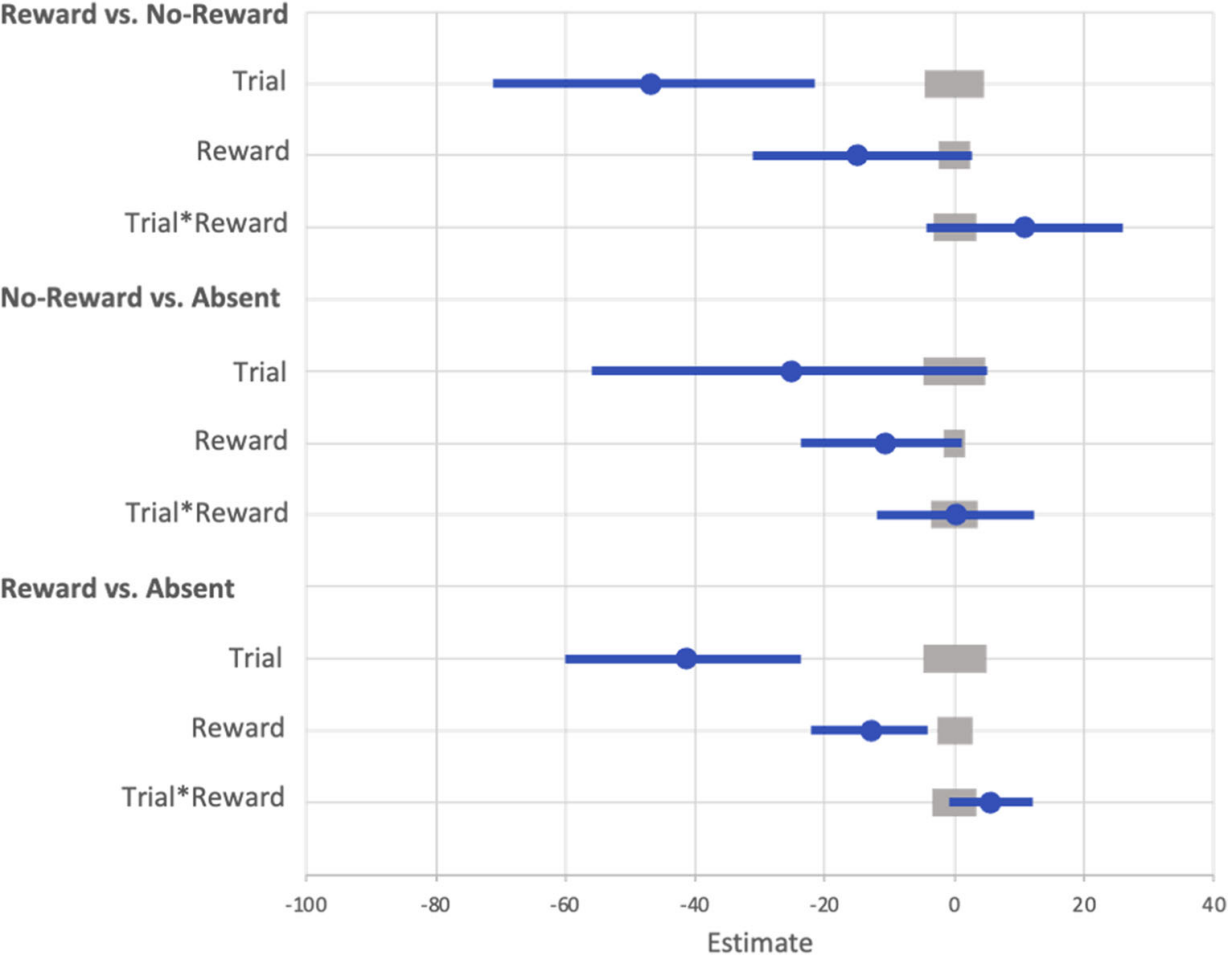
Mean point estimates and support intervals for each of the fixed effects from the generalized linear mixed effects model (GLMM) for the three comparison models during test in Experiment 4. Grey boxes denote the null region (region of practical equivalence, RPE)

The GLMM with the fixed effects of reward condition (reward vs. no-reward) and trial indicated that the effect of trial was probable (*b* = -46.88, *SI* = [-72.25, -22.61], *RPE* = ± 4.63, *BF* = 58.48; see Fig. 14). The effect of reward condition was not probable (*b* = -14.96, *SI* = [-32.55, 1.31], *RPE* = ± 2.41), and there was moderate support for the null (*BF* = 0.13). The interaction between reward condition and trial was also not probable (*b* = 10.80, *SI* = [-4.44, 25.97], *RPE* = ± 3.32), and there was decisive evidence in support of the null (*BF* < 0.01).

##### No-reward versus absent

The effect of trial was not probable (*b* = -25.26, *SI* = [-55.63, 5.40], *RPE* = ± 4.81), and there was strong evidence in support of the null (*BF* = 0.08). The effect of reward condition (no reward vs. absent) was not probable (*b* = -10.78, *SI* = [-22.64, 2.22], *RPE* = ± 1.70), and there was strong evidence in support of the null (*BF* = 0.095). The interaction between reward condition and trial was also not probable (*b* = 0.15, *SI* = [-11.89, 12.27], *RPE* = ± 3.64), and there was decisive evidence in support of the null (*BF* < 0.01).

##### Reward versus absent

The effect of trial was probable (*b* = -41.44, *SI* = [-59.23, -22.85], *RPE* = ± 4.91, *BF* = 781.95). There was anecdotal evidence of an effect of reward condition (reward vs. absent) such that participants were faster on absent trials than in the presence of formerly rewarded features (*b* = -12.92, *SI* = [-21.77, -3.80], *RPE* = ± 4.92, *BF* = 1.25). However, the interaction between reward condition and trial was not probable (*b* = 5.61, *SI* = [-0.93, 12.11], *RPE* = ± 3.46), and there was very strong evidence in support of the null (*BF* = 0.03).

### Discussion

The purpose of Experiment 4 was to assess how absent trials, i.e., the reduced frequency of exposure to the formerly reward associated feature in the absence of reinforcement, impacted the persistence of VDAC. We did not find evidence for either VDAC (reward vs. no reward) or SDAC (no reward vs. absent), or the extinction of either, and in fact found moderate evidence for the null in both cases, that is there was evidence for the lack of either a pure VDAC or SDAC effect. We did however find anecdotal evidence for an effect of VDAC+SDAC (reward vs. absent), which is to say evidence that the combination of reward-association and selection history of a feature did affect performance. However, there was no evidence that this effect was subject to extinction, as there was strong evidence for the null for the interaction.

We cannot exclude the possibility that the reduced number of learning trials may account for the lack of support for pure VDAC/SDAC effects. However, we note that the number of trials is in line with those previously reported in other VDAC experiments (e.g., VDAC effects have been reported with only 240 training trials; Anderson et al., 2011b), and that the proportion of extinction trials to training trials is the same as that in the other experiments reported here. This means that the factor of “trial” in the model is conceptually equivalent across all five experiments.

The inclusion of absent trials, which in effect reduced the probability of the presence of a reward-associated feature (as a distractor), was not sufficient to produce VDAC, nor SDAC when the reward-associated/formerly selected feature was task-irrelevant at test. However, the inclusion of absent trials did function as a useful control condition. This condition allowed us to observe that when the reward-associated feature is task-irrelevant, it is able to capture attention, but only when combined with the effects of selection. Furthermore, it appears somewhat resistant to extinction unlike VDAC alone, as we saw in Experiments 1 and 3, although the inclusion of the absent trials themselves may also have contributed to the effect due to the reduced probability of the reward-associated feature.

In Experiment 4 we also found substantial evidence in support of the null (i.e., the lack of a VDAC effect). The evidence favoring the lack of a VDAC effect paralleled the results of Experiments 2a/b. So, across three experiments when the reward associated feature was task irrelevant, but the target feature was not salient, there was evidence of varying strength from anecdotal to moderate, *against* the presence of VDAC.

## Experiment 5

In Experiment 4 the inclusion of absent trials was not sufficient to elicit VDAC in the absence of a salient singleton target at test. An interaction between reward and trial when the reward-associated feature was task irrelevant was only observed in Experiment 3 when the target was defined by a salient shape singleton. This indicates that the presence of a salient shape singleton at test may be necessary to observe an effect of reward in the typical VDAC paradigm.

In Experiment 5 we wanted to replicate the typical task features used in VDAC paradigms, the experiment was identical to Anderson and Yantis (2013) as targets were defined as the salient singleton during the test phase and there were absent trials. The motivation behind this experiment was to assess whether VDAC continued to be observed when the target was defined as a salient shape singleton at test when the frequency of exposure to the reward associated feature was reduced. The inclusion of absent trials and a non-rewarded but previously selected feature condition allowed us to obtain a measure of selection history. Although a salient singleton target was used in Experiment 3 this experiment design did not allow for the observation of selection history. In Experiment 4 where there was no salient singleton at test SDAC was not observed. It’s possible that the presence of a salient singleton during test could also lead an effect of selection history if previously selected features gain salience.

### Method

#### Participants

Participants were 39 students from the University of California Santa Barbara recruited from the research participation pool (24 female, *M*_*age*_ = 18.82 years, *SD*_*age*_ = 1.19). Four participants were excluded from the analyses because of poor performance, resulting in a final sample of 35 participants (20 female, *M*_*age*_ = 18.77 years, *SD*_*age*_ = 1.11). Participants had the opportunity to win a maximum of $8 during the learning phase and on average were paid $6.67.

#### Procedure

##### Visual search: Learning

The learning task was identical to that used in Experiment 1, except participants were given 50 practice trials, and a reduced number of learning trials – 200 rewarded and 200 non-rewarded trials compared to 400 of each in Experiment 1 (Fig. 1a). The number of learning trials in Experiment 5 were reduced for the same reasons as noted in Experiment 4.

##### Visual search: Test

During the test phase, features that were targets at learning became distractors at test, as in Experiments 2–4. In the current Experiment, targets were defined by the unique shape in the display as in Experiment 3 (Fig. 9a & b). However, unlike Experiment 3 there was a third type of condition where no previously selected target colors were presented as a feature of a distractor (absent trials). The test phase began with 20 practice trials, followed by 20 blocks each with 80 trials, yielding 400 rewarded trials, 400 non-rewarded trials and 800 absent trials.

### Results

#### Visual search: Learning

**Fig. 15:**
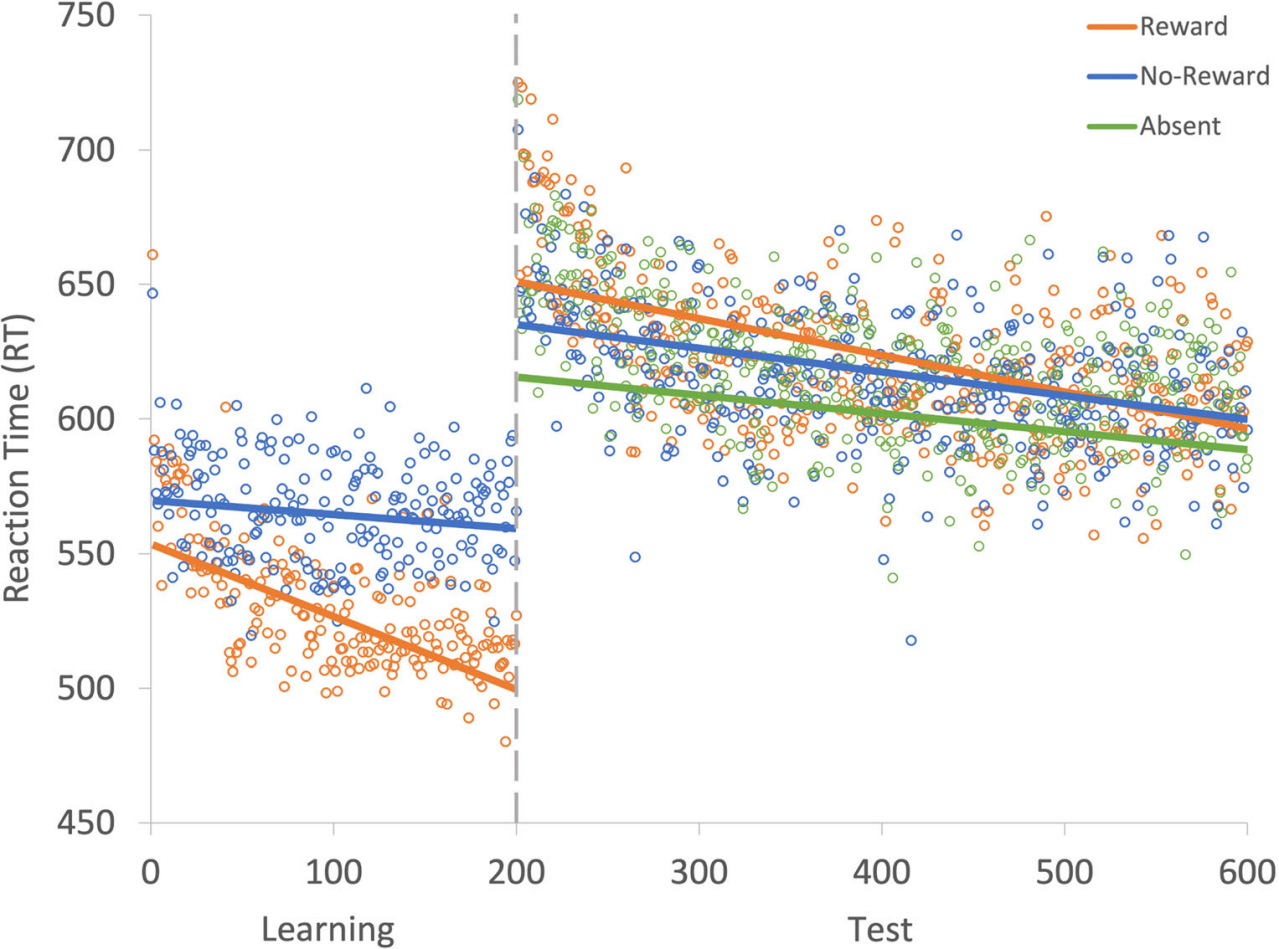
Results for both learning and the test phase of Experiment 5. Data points are the raw reaction time (RT) data averaged across participants for each trial for rewarded and non-rewarded trials separately. The regression lines are the predicted RT for rewarded and non-rewarded trials across time from the generalized linear mixed effects model (GLMM)

**Fig. 16:**
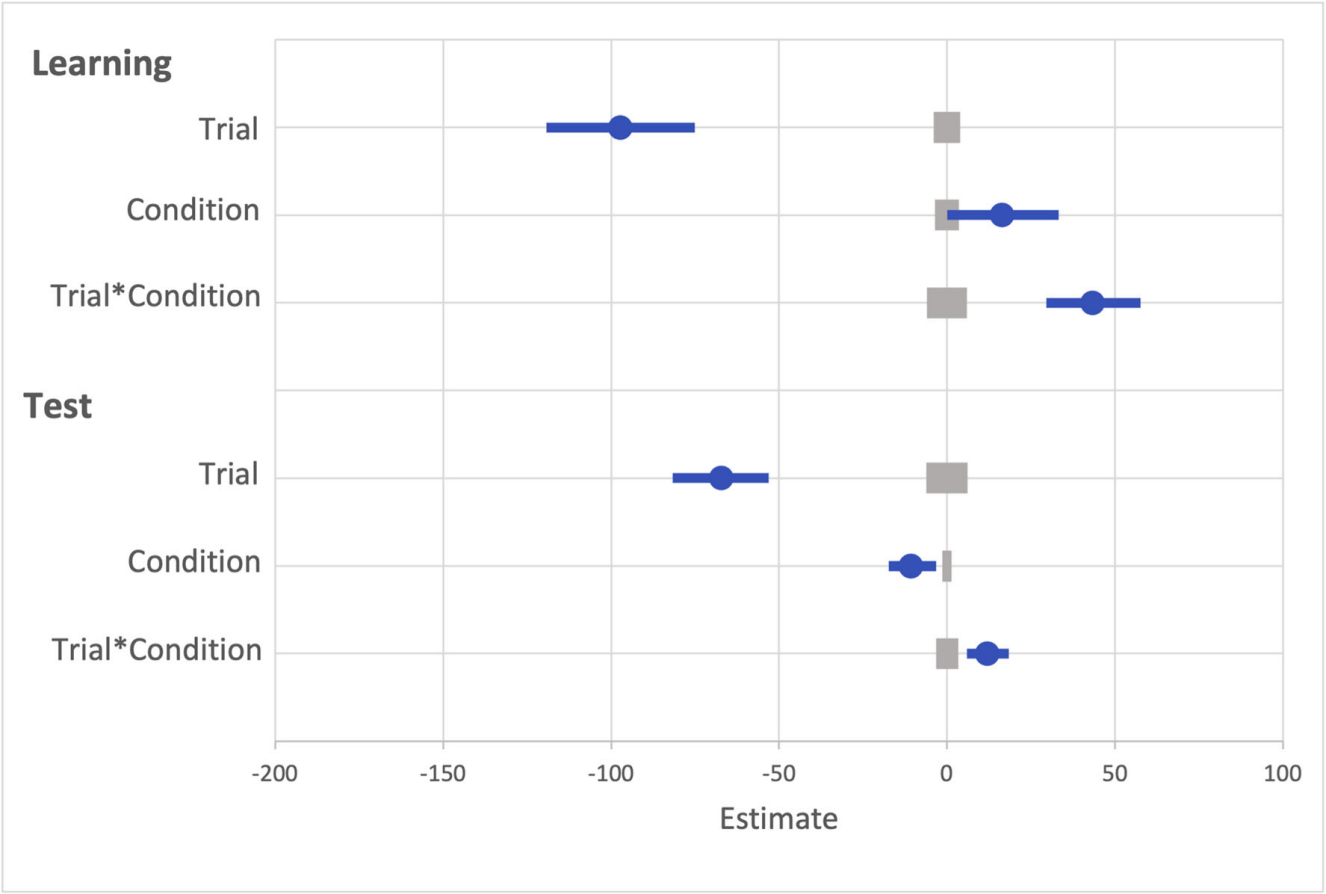
Mean point estimates and support intervals for each of the fixed effects from the generalized linear mixed effects model (GLMM) for Experiment 5 in both learning and test. Grey boxes denote the null region (region of practical equivalence, RPE)

Another GLMM was used to examine RTs on correct trials during the learning phase (see Fig. 15). The effect of trial was probable such that RTs got faster as trial order increased (*b* = -97.19, *SI* = [-119.24, -75.13], *RPE* = ± 3.97, *BF* > 1000; see Fig. 16). The effect of condition was not probable (*b* = 16.28, *SI* = [0.11, 33.19], *RPE* = ±3.57), there was substantial evidence in support of the null *BF* = 0.20 However, the interaction between trial and reward condition was probable such that RT decreased at a faster rate for rewarded than non-rewarded targets, indicating that the reward associations had been learned (*b* = 43.36, *SI* = [29.57, 57.53], *RPE* = ± 5.99, *BF* > 1000).

#### Visual search: Test

A similar GLMM was used to examine RTs on correct trials during the test phase (see Fig. 15). The effect of trial was probable such that RTs got faster as trial order increased (*b* = -67.25, *SI* = [-81.78, -52.08], *RPE* = ± 6.18, *BF* > 1000; see Fig. 16). The effect of the reward condition during the test phase was also probable (*b* = -10.71, *SI* = [-17.43, -3.32], *RPE* = ± 1.34, *BF* = 5.26). The interaction between trial and reward condition was also probable (*b* = 11.19, *SI* = [5.87, 18.02], *RPE* = ± 3.30, *BF =* 67.63).

To compare the three levels of condition (rewarded, non-rewarded, and absent) we conducted three additional GLMMs to compare reward versus no-reward, no-reward versus absent, and reward versus absent conditions.

##### Reward versus no-reward

**Fig. 17:**
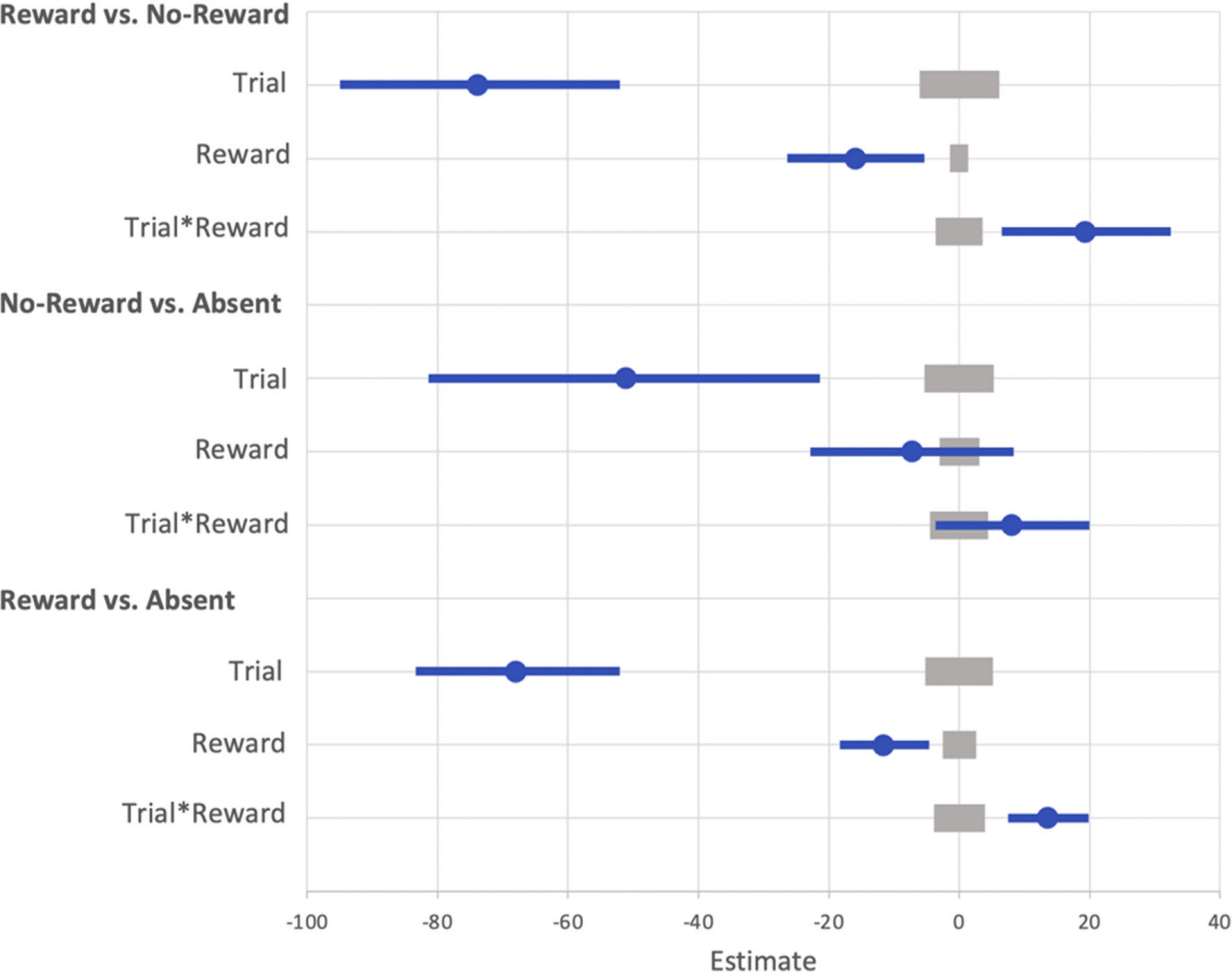
Mean point estimates and support intervals for each of the fixed effects from the generalized linear mixed effects model (GLMM) for the three comparison models during test in Experiment 4. Grey boxes denote the null region (region of practical equivalence, RPE)

The GLMM with the fixed effects of reward condition (reward vs. no-reward) and trial indicated that the effect of trial was probable such that participants became faster over the test phase (*b* = -73.89, *SI* = [-94.85, -53.33], *RPE* = ± 5.20, *BF* > 1000; see Fig. 17). The effect of reward condition was probable such that participants were slower on trials where the reward associated feature was presented compared to the non-reward associated (*b* = -15.92, *SI* = [-26.38, -5.34], *RPE* = ± 1.41, *BF =* 6.03). The interaction between reward condition and trial was also probable such that the difference in reaction time between rewarded and non-rewarded trials decreased throughout the test phase (*b* = 19.30, *SI* = [6.21, 32.15], *RPE* = ± 3.64, *BF =* 2.59).

To assess whether VDAC was completely extinguished in the test phase, a Bayesian paired-samples t-test examining the difference between rewarded and non-reward was conducted on the final 100 trials of the test phase. There was anecdotal evidence in support of the null (*BF* = 0.55) suggesting that VDAC was no longer present at the end of the test phase.

##### No-reward versus absent

The effect of trial was probable such that participants became faster over the test phase ( = -51.11, *SI* = [-80.85, -20.82], *RPE* = ± 5.29, *BF* = 11.92). The effect of reward condition (no reward vs. absent) was not probable (*b* = -7.19, *SI* = [-22.68, 8.44], *RPE* = ± 3.08), there was very strong evidence in support of the null (*BF* = 0.015). The interaction between reward condition and trial was also not probable (*b* = 8.01, *SI* = [-3.91, 19.63], *RPE* = ± 4.45), and there was strong evidence in support of the null (*BF* = 0.033).

##### Reward versus absent

The effect of trial was probable such that participants became faster over the test phase (*b* = -67.99, *SI* = [-83.86, -52.61], *RPE* = ± 5.20, *BF* > 1000). The effect of reward condition (reward vs. absent) was probable such that participants were slower in the presence of formerly reward associated feature compared to absent trials (*b* = -11.62, *SI* = [-18.65, -4.98], *RPE* = ± 2.54, *BF* = 17.57). The interaction between reward condition and trial was also probable (*b* = 13.60, *SI* = [7.32, 19.66], *RPE* = ± 3.90, *BF* = 218.98), suggesting that the difference between rewarded and absent trials decreased throughout the test phase.

To assess whether the difference between reward and absent trials was still present at the end of the test phase. A Bayesian paired-samples t-test examining the difference between rewarded and absent trials on the final 100 trials revealed that there was substantial evidence in support of the null (*BF* = 0.25) suggesting that the effect of both VDAC and SDAC was no longer present.

### Discussion

The purpose of Experiment 5 was to assess whether the VDAC would be observed with the inclusion of absent trials and a physically salient target. In the current experiment it was possible to examine the effects of selection history in the presence of a physically salient singleton but no evidence of a purely selection history driven effect was found. However, we did find evidence for VDAC consistent with Anderson & Yantis (2013). These results suggest that for VDAC to be observed both a physically salient target must be present during test and frequency of exposure to the reward-associated feature must be reduced. This supports the possibility that when searching for a target defined as a physically salient singleton, salient features gain weight across dimensions, including both the target and the reward-associated distractor, and this, at least when complicated by inter-dimension competition, is sufficient for VDAC when the reward-associated feature is task-irrelevant (e.g., Bacon & Egeth, 1994). The inclusion of absent trials may have also contributed to the observation of VDAC in the current experiment. The reduced exposure to the reward-associated feature in Experiment 5 may have increased the time to learn the association between the reward-associated feature and the absence of reward which resulted in the main VDAC effect being observed in this experiment, but not in Experiment 3. These findings are consistent with findings showing that infrequent distractors are less effectively ignored than frequent ones (Geyer et al., 2008; Müller et al., 2009).

The VDAC effect was found to decrease over time and was no longer present in the final 100 trials of the test phase suggesting that VDAC is subject to extinction. Furthermore, a similar pattern of results to Experiment 3 can be seen as RTs to rewarded trials become faster relative to non-rewarded trials suggesting that the reward-associated distractor may be suppressed over time to avoid distraction. These results contrast with Anderson and Yantis (2013) who report a lack of extinction of VDAC in the absence of reinforcement. Therefore, we failed to replicate that VDAC results in an enduring change in attentional priority even when using identical task features.

We also found evidence of an impact of the combination of both VDAC and SDAC but no pure selection driven effect consistent with results in Experiment 4. In the context of the current experiment and the inclusion of the salient shape singleton target, these findings could suggest that reward associated features gain salience across both feature dimensions leading to VDAC. However, previously selected features are not subject to the same gains is salience as previously reward associated features, as SDAC was not observed even when a physically salient singleton is present.

## General discussion

VDAC is an involuntary bias of attention for a feature that was predictive of a rewarding outcome, in the absence of reinforcement of the reward association (Anderson et al., 2011b; Anderson & Yantis, 2013). In the present study, we investigated how typical design features of VDAC paradigms affect the persistence of VDAC, and its extinction. In all experiments, during the learning phase, there was either an effect of reward condition and/or an interaction between reward condition and trial order, which indicated that participants learned the feature-reward contingencies. The reliable observation of reinforcement learning here is in contrast to many reports where VDAC was observed at test without evidence of reinforcement learning during the learning phase (Mine & Saiki, 2015; Rajsic et al., 2017; Roper et al., 2014; Sali et al., 2018; Theeuwes & Belopolsky, 2012). During the test phase it was found that VDAC task-features affect whether VDAC is observed or not, VDAC was only reliably observed when there was a salient singleton target and absent trials were present which are the typical design features. Therefore, to observe VDAC it may be necessary to have the combination of the salient singleton and absent trials as both factors are known to increase vulnerability to capture. When VDAC was observed it was subject to extinction suggesting that reward-associations do not create an enduring change in attentional priority and can be overcome when the reward-associated distractor is presented in the absence of reward over many trials.

### Presence of value-driven attention capture

Evidence of an effect of reward was observed in two cases in the test phases of the current study: either when (1) the formerly reward-associated feature remained task relevant (Experiment 1), or (2) the target feature was physically salient and orthogonal to the task-irrelevant, reward-associated feature and there was a reduced frequency of exposure to the reward-associated feature (Experiment 5). Indeed, when the reward associated feature was task-irrelevant at test, as is typical of VDAC paradigms, we found evidence against the presence of VDAC in four separate experiments (Experiments 2a, 2b, 3, and 4).

The only experiment where we reliably found evidence of VDAC was in Experiment 5, which was a direct replication of typical VDAC design features (a physically salient target and the inclusion of absent trials). This indicates that the combination of these design features may be necessary to give rise to VDAC. VDAC was also not persistent throughout the test phase suggesting that VDAC does not create an enduring change in attentional priority and is very much subject to extinction which is inconsistent with previous claims (e.g., Anderson & Yantis, 2013). In Experiment 3 we did find evidence of an extreme interaction between reward condition and trial which indicated that reward did have an impact on attention as trails with the reward-associated feature present became faster over time relative to trials with the non-reward associated feature, an effect that was similarly observed in Experiment 5.

As described earlier, the current study was not intended to be an exhaustive investigation of the boundaries of the VDAC effect, its persistence, or extinction. However, our study does investigate, and indeed support, the possibility that common paradigm design features, not typically described as being particularly critical, are in fact important for the persistence of VDAC. One feature appears to be critical, and there are good reasons for that to be so. Specifically, to make the reward-associated feature irrelevant at test the target feature is often orthogonal to the reward-associated feature, i.e., from a different feature dimension and also physically salient. Our results show that this choice is not benign. The implications for this result are discussed further in the following section. The inclusion of absent trials in Experiment 5 could have also contributed to the observation of VDAC as the control of attention is affected by the frequency of distractors such that infrequent distractors are less effectively ignored than frequent ones (Geyer et al., 2008; Müller et al., 2009). The combination of both factors could be necessary to observe to VDAC.

### Extinction of value-driven attention capture

In addition to the persistence of VDAC we also investigated the extinction of VDAC – that is, the reduction of the VDAC effect with repeated exposures in the absence of reinforcement (Pavlov, 2010). VDAC extinction, or lack thereof, is not often reported (but see Anderson et al., 2011a, 2016; Asutay & Västfjäll, 2016; Sali et al., 2018). As discussed earlier, VDAC has been observed following both Pavlovian (Bucker & Theeuwes, 2017) and instrumental learning (Failing & Theeuwes, 2017). In either case, if VDAC is an example of such conditioning, one would expect to observe its extinction. If VDAC does not extinguish, it suggests that conditioning alone does not result in the acquisition or persistence of VDAC, but rather that there is some additional factor that uniquely prevents typical extinction learning. We found evidence of extinction of a reduction in the effect of reward in both cases where there where there was an effect of reward during test in the absence of reinforcement when the formerly reward associated feature: (1) remained task relevant (Experiment 1); and (2) was task irrelevant, but was orthogonal to the target feature, which itself was physically salient (Experiment 5). Evidence for extinction of the effect of reward was anecdotal in the case of Experiment 1, where the reward-associated feature remained task relevant at test (during extinction learning). The evidence of an interaction between reward and trial was extreme in the case of Experiments 3 and 5, where the reward-associated feature was task irrelevant at test, in the context of a salient and orthogonal target feature. Experiment 5 is closest to the typical VDAC paradigm, and the results indicate that VDAC is very much subject to extinction. The fact that evidence of a reduction in the effect of reward in Experiment 1 was only anecdotal may be due to the interference to extinction learning where the conditioned stimulus (reward-associated feature) must still be attended, although other possibilities exist and deserve to be investigated further. While our results are not conclusive as to what factors influence the extinction of VDAC in the absence of reinforcement, they do allow us to conclude that: (1) VDAC is subject to extinction, and (2) whether VDAC is extinguished may depend on whether the reward-associated feature is unattended, and (3) also possibly whether it is outcompeted for visual attention by an orthogonal and physically salient target feature (Treisman, 1985; Wolfe, 1994). One possible explanation of why the presence of the singleton increases the likelihood of extinction of VDAC is that the conspicuity of the target facilitates the resolution of the competition between the previously rewarded associations and the new associations. Capture by the reward-associated feature may be actively suppressed when using singleton search due to such competition. We observed that trials containing the reward-associated feature became faster relative to the non-rewarded trails in both Experiments 3 and 5 consistent with the suggestion that the reward-associated feature is suppressed over time to avoid distraction.

Furthermore, the length of our test phases were 1600 trials with 800 and 400 exposures to the reward associated feature in Experiment 3 and 5 respectively. In contrast, previous work has used between 60 and 120 trials with a reward-associated feature during the test phase (Anderson et al., 2011a, b; Anderson & Yantis, 2012, 2013). It’s likely that previous studies did not have a sufficient number of trials to observe extinction. However, our results demonstrate that over many exposures in the absence of reward that VDAC is subject to extinction.

### Selection demands

The key pattern of evidence emerging from the current study is that it appears that when the reward-associated feature is task-irrelevant at test, VDAC depends on how the target is defined. Specifically, we only observed VDAC in the presence of a salient shape singleton target and the inclusion of absent trials. As mentioned earlier, this difference in how the target is defined amounts to a difference in selection demands of the visual search task at test. In Experiments 2a/b/4 the target could not be selected based on salience and *required* the distinction between kinds (or feature values) of the same feature dimension. In contrast, in the two Experiments where there was evidence of an effect of reward when task irrelevant (Experiments 3 and 5), the visual search task at test was a singleton search that demanded the most salient feature of a specific dimension be selected and did not require the discrimination within that dimension. Arguably, this task also did not demand that salient features from other dimensions be ignored. In contrast to the other experiments reported here, the target was not defined by contrast with another feature or dimension, rather the target set was the most salient shape (i.e., the strongest signal in salience map for shape). Although the ability to ignore salient features from other dimensions may affect performance on that task and appears to in the case where there is evidence of VDAC. This explanation is consistent with previously reported differences between singleton search and feature search strategies showing larger capture effects during search for targets defined as the odd-one-out in a display compared to search for targets defined by a specific feature (or combination of features; e.g., Bacon & Egeth, 1994; Lamy et al., 2006; Lamy & Egeth, 2003).

If the presence of VDAC requires singleton search, then why has VDAC been observed in tasks that do not demand singleton search (e.g., Della Libera & Chelazzi, 2009; Failing & Theeuwes, 2014; MacLean & Giesbrecht, 2015a, b)? It is important to note that the tasks used in these studies were either not visual search (Della Libera & Chelazzi, 2009; Failing & Theeuwes, 2014), or were visual search with partial report (MacLean & Giesbrecht, 2015a, b). These tasks make very different demands than the visual search task typically used in VDAC experiments, and it appears that the type of search demanded by the task plays a very important role in whether VDAC is observed or not.

One possible explanation for this seemingly discrepant pattern of results is the nature of the task-relevant attentional set. In each of the studies that observed VDAC in the absence of a target defined by a salient singleton, the potential target set was greater than one. A focused, or singular, attentional set may prevent distraction by the reward-associated feature, while a wider set (>1) creates a vulnerability to capture or distraction. When searching for one specific target item, as is the case with the shape singletons in typical VDAC experiments (and the present Experiments 3 and 5), all other features/dimensions can be ignored. When search involves a greater set, more features/classes must be attended, thereby increasing the likelihood that an irrelevant feature/dimension captures attention. Importantly, if this is the case, then it appears that the larger attentional set must also consist of a different class (or dimension/category) than the previously rewarded feature, in the absence of a salient target; at least in the context of a visual search task as used here, and as is typical of VDAC experiments. This is because in Experiments 2a/b/4, the target set was >1 (two colors), but the target set and the previously rewarded feature were the same feature class (i.e., color). More generally this means that VDAC occurs more reliably when there is some vulnerability introduced by the search mode, either by salience, a wider attentional set, and/or inter-class competition. The vulnerability required to elicit VDAC is likely task dependent. Further work would be required to test this possibility; however, it does suggest that VDAC is not unavoidable and can be prevented quite effectively with a narrow focus of attention as demanded by the task.

### Selection-driven attention capture (SDAC)

In addition to reward, selection history – previous experience selecting a feature – appears to play a critical role in shaping attention, unique from that of reward history. In the current study, two experiments allowed us to explore the persistence of SDAC and its extinction – Experiments 4 and 5, where the use of absent trials provides a baseline for comparison with trials where a former target feature was present (non-rewarded), and where a former target and former reward-associated feature was present (rewarded). We did not find evidence that selection history resulted in persistent SDAC in the absence of reinforcement or selection. However, the combination of selection and reward history’s effects on performance was a unique case among those experiments where no evidence of VDAC was observed. It appears that while reward and selection history alone are not sufficient to produce effects in some cases, the combination of the two may, although possibly only when the presence of the reward-associated feature is relatively rare. This provides further support for the premise that attention is not driven only by current goals but also by several features that have guided our attention in the past (Awh et al., 2012). However, it also reinforces the need to consider that the interaction of selection history with reward history may contribute to VDAC when observed using reward-associated features that were also previously selected.

### Predictors of value-driven attention capture persistence

Finally, we failed to replicate that individual differences in VWM capacity could predict VDAC inconsistent with previous findings (Anderson et al., 2011b). Previous evidence has shown that VDAC is predicted by individual differences in visual working memory (VWM) capacity (Anderson et al., 2011b). However, none of the experiments reported here employ a paradigm identical to that used in the context where the relationship was previously reported (Anderson et al., 2011b). It is possible that the relationship between VWM capacity and VDAC is particular to that paradigm. Furthermore, we found evidence that BIS was associated with VDAC during Experiment 1, such that those who have a higher BIS score also had a larger capture effect. However, these correlations were not replicated in any of the other experiments. We should also note that the sample sizes we have in each experiment may not have been sufficient to detect such relationships.

## Conclusions

Overall the findings from the current study indicate that attention can be captured by features that have guided our attention in the past and features previously associated with reward, providing further evidence that the top-down and bottom-up dichotomy does not sufficiently account for the factors determining priority for attention (Awh et al., 2012). However, our findings indicate that observing VDAC depends on the design of the VDAC paradigm used. Specifically, the use of a salient and orthogonal target feature may increase the likelihood of capture because of the type of search strategy employed in these tasks. In addition, the inclusion of absent trials may further enhance the effects of a reward-associated distractor when the target feature is salient as less frequent distractors are more difficult to ignore. We only reliably found evidence of VDAC in Experiment 5 which used identical features typically used in VDAC visual search tasks suggesting these typical features may be a necessary task design to observe VDAC. Furthermore, we found that VDAC in Experiment 5 did not persist in the absence of reinforcement which is inconsistent with reports that VDAC creates an enduring change in attentional priority. Given the homogeneity of the paradigms used when drawing conclusions about VDAC this has implications for a large part of the literature

## Footnotes

1 These correlations were not replicated in any of the subsequent experiments reported here.

2 There was also no evidence for correlations in Experiments 2–4. However, there was anecdotal evidence of a correlation between VDAC (reward vs. no reward RT) and working memory capacity in Experiment 5 (*r*(33) = -.39, *BF* = 2.74). Thus, we failed to reliably replicate the relationship between VWM and VDAC previously observed in any of the five Experiments, despite observing VDAC. The lack of evidence for an effect may be the result of insufficient power to detect the correlation (although *n* = 24–26 in Anderson et al., 2011b). However, when we combined the samples across Experiments where there were equivalent conditions (the difference between reward and no-reward when on a distractor i.e., Experiments 2–5; *M* = 3.07, *SD* = 1.00), we still did not find evidence of a correlation (*r*(109) = -0.13, *BF* = 00.31). Another possibility is that our definition and calculation of VDAC differs from Anderson et al., and thus we did not replicate the effect as we did not replicate the calculation of VDAC. We define VDAC as the difference in reaction time in the presence of a reward associated distractor, and in the presence of a non-reward associated distractor. However, the correlation reported by Anderson et al. was between VWM capacity and the difference in reaction time in the presence of a high reward-associated distractor and the *absence of any reward-associated distractor*. When we calculated VDAC as Anderson et al., however, we still did not find sufficient evidence for a correlation (Experiments 4 and 5; *r*(51) = -.24, *BF* = 0.70).
